# Genetically encoded formaldehyde sensors inspired by a protein intra-helical crosslinking reaction

**DOI:** 10.1038/s41467-020-20754-4

**Published:** 2021-01-25

**Authors:** Rongfeng Zhu, Gong Zhang, Miao Jing, Yu Han, Jiaofeng Li, Jingyi Zhao, Yulong Li, Peng R. Chen

**Affiliations:** 1 Synthetic and Functional Biomolecules Center, Beijing National Laboratory for Molecular Sciences, College of Chemistry and Molecular Engineering, Peking University, 100871 Beijing, China; 2 Peking-Tsinghua Center for Life Sciences, 100871 Beijing, China; 3 State Key Laboratory of Membrane Biology, PKU-IDG/McGovern Institute for Brain Research, School of Life Sciences, Peking University, 100871 Beijing, China; 4 Key Laboratory of Bioorganic Chemistry and Molecular Engineering of Ministry of Education, Peking University, 100871 Beijing, China; 5 Present Address: State Key Laboratory of Cellular Stress Biology, Innovation Center for Cell Signaling Network, School of Life Sciences, Xiamen University, 361005 Xiamen, Fujian China; 6 Present Address: Chongqing Key Laboratory of Natural Product Synthesis and Drug Research, School of Pharmaceutical Sciences, Chongqing University, 401331 Chongqing, China; 7 Present Address: Chinese Institute for Brain Research, 102206 Beijing, China

**Keywords:** Transcription factors, Fluorescent proteins, DNA-binding proteins, X-ray crystallography

## Abstract

Formaldehyde (FA) has long been considered as a toxin and carcinogen due to its damaging effects to biological macromolecules, but its beneficial roles have been increasingly appreciated lately. Real-time monitoring of this reactive molecule in living systems is highly desired in order to decipher its physiological and/or pathological functions, but a genetically encoded FA sensor is currently lacking. We herein adopt a structure-based study of the underlying mechanism of the FA-responsive transcription factor HxlR from *Bacillus subtilis*, which shows that HxlR recognizes FA through an intra-helical cysteine-lysine crosslinking reaction at its N-terminal helix α1, leading to conformational change and transcriptional activation. By leveraging this FA-induced intra-helical crosslinking and gain-of-function reorganization, we develop the genetically encoded, reaction-based FA sensor—FAsor, allowing spatial-temporal visualization of FA in mammalian cells and mouse brain tissues.

## Abstract (web summary)

In order to understand the role of formaldehyde in living systems, real-time monitoring is required. Here the authors report a genetically encoded, reaction-based formaldehyde sensor (FAsor) that enables visualisation of formaldehyde in mammalian cells and tissues.

## Introduction

In addition to its broad presence in environment, various cellular processes such as the one-carbon metabolism and the oxidative demethylation on proteins and nucleic acids produce formaldehyde (FA)^1–3^. As the simplest reactive carbonyl species (RCS), the cytotoxicity of FA has been well documented due to its high reactivity with various biological nucleophiles^4–6^. Meanwhile, the high physiological abundance of FA (e.g., ~0.1 mM in blood, and 2–4 times higher in liver and nasal mucosa) indicates potential physiological effects that have been increasingly appreciated^7–9^. For example, low concentration of FA is reported to increase human melanoma cell proliferation and MAPK pathway activation^10,11^. Additionally, FA is found to support the survival of one-carbon-cycle-defective cells recently^12^. In methylotrophic bacteria, FA even serve as a sole carbon source, being assimilated via ribulose monophosphate (RuMP) pathway^13^. A major challenge in deciphering the biological roles of FA is the real-time measurement of its concentration, duration, and location in living cells and tissues. Although FA is an abundant molecule in vivo, stringent protection mechanisms have been evolved to scavenge this highly reactive RCS with little knowledge about its intracellular distribution^14–16^. Fluorescent sensors represent a powerful noninvasive tool for real-time monitoring of endogenous FA organization in space and time and small molecule-based FA sensors have been elegantly developed in recent years^17–22^. Alternatively, genetically encoded, fluorescent protein (FP)-based sensors are advantageous for subcellular targeting and cell-type-specific expression as well as potential long-term imaging in animals^23–25^. However, no such genetically encoded FA sensors are currently available.

A major hurdle is due to the lack of knowledge and molecular details on whether and how a protein can actively recognize and transduce the “signal” from a highly reactive FA molecule, which could be converted into a fluorescence change on the coupled FPs^26^. To this end, we first study the structural and molecular mechanism of a FA-responsive transcription factor HxlR^27^, which reveals a FA-triggered crosslinking reaction between the side-chains of residues Cys11 and Lys13 on helix α1. The resulting intrahelical methylene bridge (Cys-CH_2_-Lys) represents a protein modification with a conformational change that allosterically induced HxlR’s transcriptional activation. Inspired by the unique gain-of-function FA-sensing mechanism of HxlR, we convert its intrahelical crosslinking-induced conformational change into a fluorescence change of the inserted circularly permutated yellow FP (cpYFP). This genetically encoded FA sensor—FAsor allows direct visualization of FA in diverse living species including mammalian cells as well as the brain tissue of mice.

## Results

### FA directly activates HxlR via a gain-of-function, intrahelical crosslinking reaction

Among the currently reported putative FA-responsive proteins we surveyed, *Bacillus subtilis* HxlR is particularly intriguing because this MarR/DUF24 family transcriptional regulator “positively” responds to FA with an enhanced transcription activity^27,28^ (Supplementary Note 1, Supplementary Fig. 1a). Such a gain-of-function response implies an active recognition and transducing mechanism of the FA signal by HxlR. In contrast, for the FA transcriptional factors that are inactivated by FA, it is difficult to exclude the possibility of damaging effects as opposed to specific recognition^26,29,30^. We thus decided to adopt a structure-based approach to study this positive FA regulator.

**Fig. 1:**
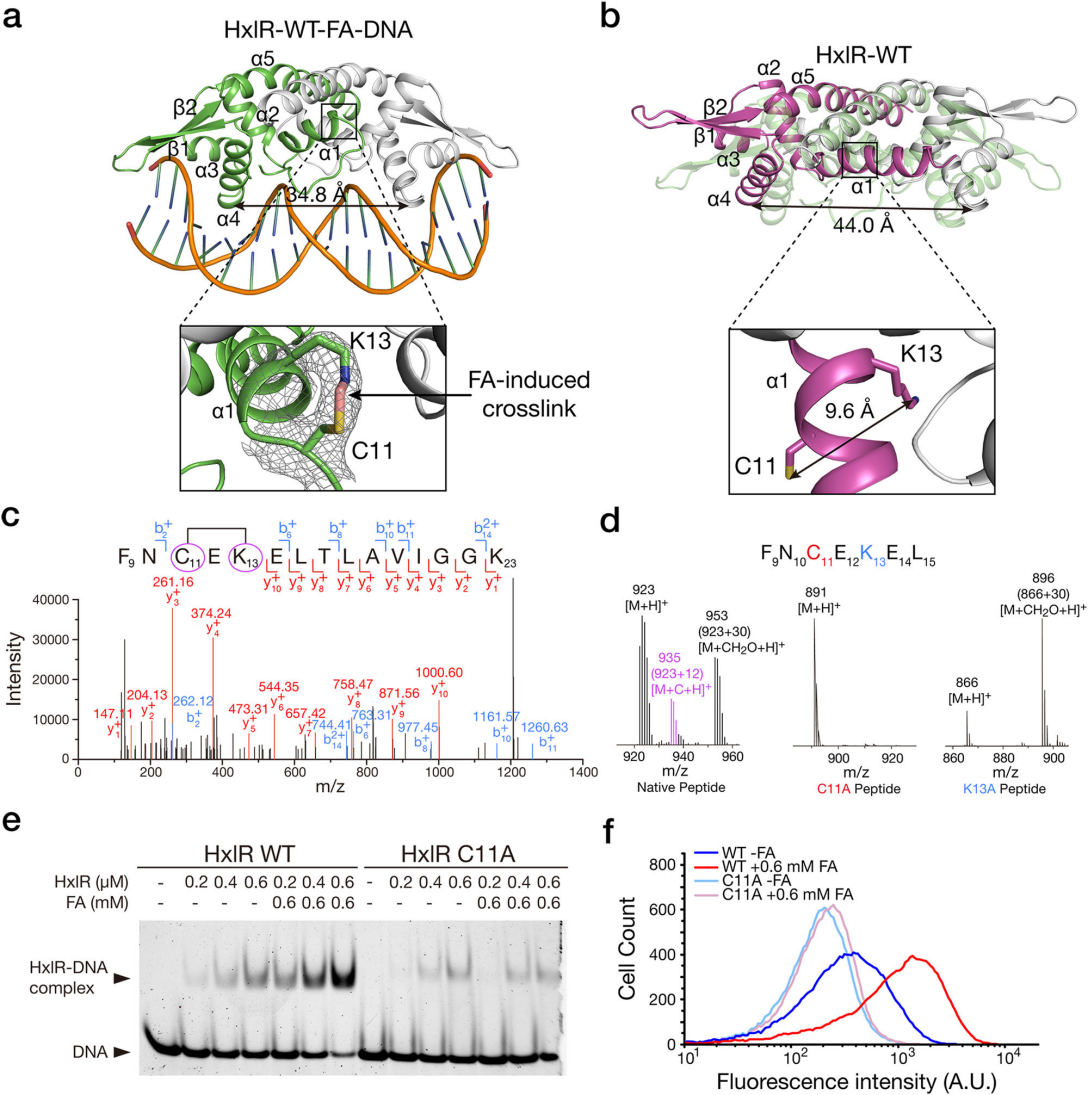
HxlR directly senses FA via the intrahelical Cys-Lys crosslinking reaction. **a** Crystal structure of FA-activated HxlR protein/DNA complex (HxlR-WT-FA-DNA). Close-up view (right) shows the FA-triggered crosslinking reaction between residues Cys11 and Lys13 on helix α1. The 2*F*_o_-*F*_c_ electron density map contoured at 1*σ* indicates that an intrahelical methylene bridge (pink) was formed between Cys11 and Lys13 upon FA treatment. **b** Crystal structure of HxlR protein in the absence of FA (HxlR-WT). Close-up view (right) shows the side-chains of residues Cys11 and Lys13 which face opposite directions. The distance between the thiol group on Cys11 and the amine group on Lys13 is 9.6 Å, indicating no interactions between these two residues. Superposed structures of HxlR-WT and HxlR-WT-FA-DNA (light green) showed obvious conformational change with FA-induced intrahelical crosslinking reaction. **c** MS-MS characterization of FA-induced methylene bridge between Cys11 and Lys13 on intact HxlR protein under living conditions. In the MS/MS spectrum of a peptide F9-K23, the fragment ions b2^+^ and y10^+^ correspond to the peptide fragments _9_FN_10_ and _14_ELTLAVIGGK_23_, respectively (observed *m/z* 262.12 and 1000.60), which verified the methylene bridge formation between Cys11 and Lys13 (+12 Da, highlighted by purple circles and the black line between them). **d** LC-MS analysis verifying the FA-induced methylene bridge between Cys11 and Lys13 on a peptide sequence derived from HxlR’s helix α1. Mutation of either Cys11 or Lys13 abolished the methylene bridge formation (+12 Da, the [M + C + H]^+^ ion). **e** Cys11 is essential for FA-enhanced DNA binding of HxlR. Determination of HxlR’s DNA-binding ability via EMSA showed that 0.6 mM FA was able to increase DNA-binding ability of HxlR-WT protein, whereas little change was observed for the HxlR-C11A mutant, in the presence of FA. **f** Flow cytometric analysis of the *hxlAB*-*gfp* reporter-harbored *E. coli* BW25113 cells expressing HxlR-WT or HxlR-C11A proteins with and without FA treatment. The addition of 0.6 mM FA increased the expression level of GFP in the bacterial strain expressing HxlR-WT, while little change was observed on the bacterial strain expressing HxlR-C11A, indicating that Cys11 is essential for FA-enhanced transcription activity of HxlR in vivo.

We first solved the crystal structures of HxlR with and without FA. After optimizing the DNA sequence, we successfully crystallized the HxlR-DNA complex after FA treatment and the resulting structure, HxlR-WT-FA-DNA, was solved to 2.9-Å resolution (Fig. 1a and Supplementary Table 1, Supplementary Note 2). The crystal structure of wild-type HxlR without DNA and FA treatment (HxlR-WT) was also solved to 2.6-Å resolution (Fig. 1b and Supplementary Table 1). Both FA-treated and -untreated HxlR adopt an overall topology similar to the previously reported MarR/DUF24 family proteins that bind DNA in a dimeric form, with each monomer consisting of dimerization domain (helices α1 and α5) and winged helix-turn-helix (wHTH) DNA-binding domain (helices α2, α3, α4, and β-strands)^31–33^. The distance between the DNA-binding helices α4 and α4′ were 34.8 Å in HxlR-WT-FA-DNA and 44.0 Å in HxlR-WT, consistent with the DNA-bound and non-DNA-bound forms of MarR/DUF24 family proteins, respectively (Fig. 1a). Interestingly, a covalent bridge was clearly visible between the side-chains of Cys11 and Lys13 on the 2*F*_o_-*F*_c_ electron density map of the HxlR-WT-FA-DNA structure (Fig. 1a), while the side-chains of Cys11 and Lys13 were located oppositely in helix α1 and their side-chains were separated 9.6 Å away from each other in the HxlR-WT structure (Fig. 1b). Further refinement of the HxlR-WT-FA-DNA structure modeled a methylene bridge connecting the thiol group on Cys11 and the amine group on Lys13 (Cys-CH_2_-Lys; Fig. 1a). Particularly, Cys11 is a conserved residue and has been reported to react and sense electrophiles and/or oxidants in other MarR/DUF24 family members (Supplementary Fig. 1b)^32–34^. We thus speculated that HxlR may react with FA via Cys11 and Lys13, and the resulting intrahelical methylene bridge and conformational changes may allosterically regulate its DNA-binding domain.

In order to verify our speculations, we first used mass spectrometry (MS) to validate the FA-induced methylene bridge between N-terminal Cys11 and Lys13 observed in the crystal structure. Our FT-MS analysis of the purified HxlR protein before and after FA treatment showed a newly formed +12 Da mass peak in the FA-treated sample, corresponding to formation of a methylene bridge in the protein (Supplementary Fig. 1c). Next, we stimulated the HxlR-expressing cells with FA and performed MS-MS analysis on the purified HxlR protein. Consistent with the results on the purified protein, a +12 Da mass deviation was found on MS1 scan of the peptide containing Cys11 and Lys13, and the MS2 spectra of the same peptide further verified a methylene bridge formation between these two residues (Fig. 1c). Finally, we verified the FA-induced Cys11-Lys13 crosslinking reaction and the methylene bridge formation via a synthetic peptide containing the native residues from Phe9 to Leu15 on HxlR protein (Ac-FNCEKEL-NH_2_). The newly formed +12 Da mass peak on the peptide after FA incubation, which was absent in the peptides with Ala replacing Cys11 or Lys13, confirmed that FA is able to induce intramolecular crosslinking with a methylene bridge formation between Cys11 and Lys13 in this peptide (Fig. 1d, Supplementary Note 3 and Supplementary Fig. 1d).

We next investigated whether the FA-triggered intrahelical crosslinking reaction could lead to HxlR activation. First, we performed an in vitro electrophoretic mobility shift assay (EMSA) to compare HxlR’s DNA-binding ability with and without FA treatment. The results showed that incubating HxlR-WT with 0.6 mM FA was able to enhance its DNA affinity (Fig. 1e). We then generated HxlR mutants with Cys11 mutated to Ala (HxlR-C11A), and utilized the same assay to evaluate the effect of FA on their DNA binding. Different from HxlR-WT, HxlR-C11A mutant exhibited little change after incubation with the same amount of FA (Fig. 1e), indicating that FA can specifically activate HxlR through the intrahelical Cys-Lys crosslinking reaction. We next validated this FA-induced HxlR activation in vivo by monitoring the transcription activity. A HxlR-inducible GFP reporter encoding the *gfp* gene under the control of *hxlAB* promoter (*hxlAB-gfp*) was transformed into the *E. coli* BW25113 strains expressing HxlR-WT and HxlR-C11A, respectively (Supplementary Fig. 2a)^27^. Having confirmed that FA affected viability of bacteria little in the measurement, we treated the bacterial cells with or without FA, followed with analyzing the viability and HxlR-regulated transcription activity via flow cytometry (Supplementary Fig. 2b, c). Indeed, FA was able to increase *gfp* expression under the control of HxlR-WT, but not HxlR-C11A mutant (Fig. 1f). In addition, we measured the effect of K13A mutation on HxlR’s DNA binding and transcriptional regulation. Although with a higher basal level on DNA binding and transcriptional activation, the K13A mutant (HxlR-K13A) also remained insensitive to FA (Supplementary Fig. 2d, e). Taken together, we confirmed that HxlR uses a gain-of-function, intrahelical crosslinking mechanism between its N-terminal residues Cys11 and Lys13 to sense FA and trigger the transcriptional responses.

### Structural bases for FA-induced HxlR activation

**Fig. 2:**
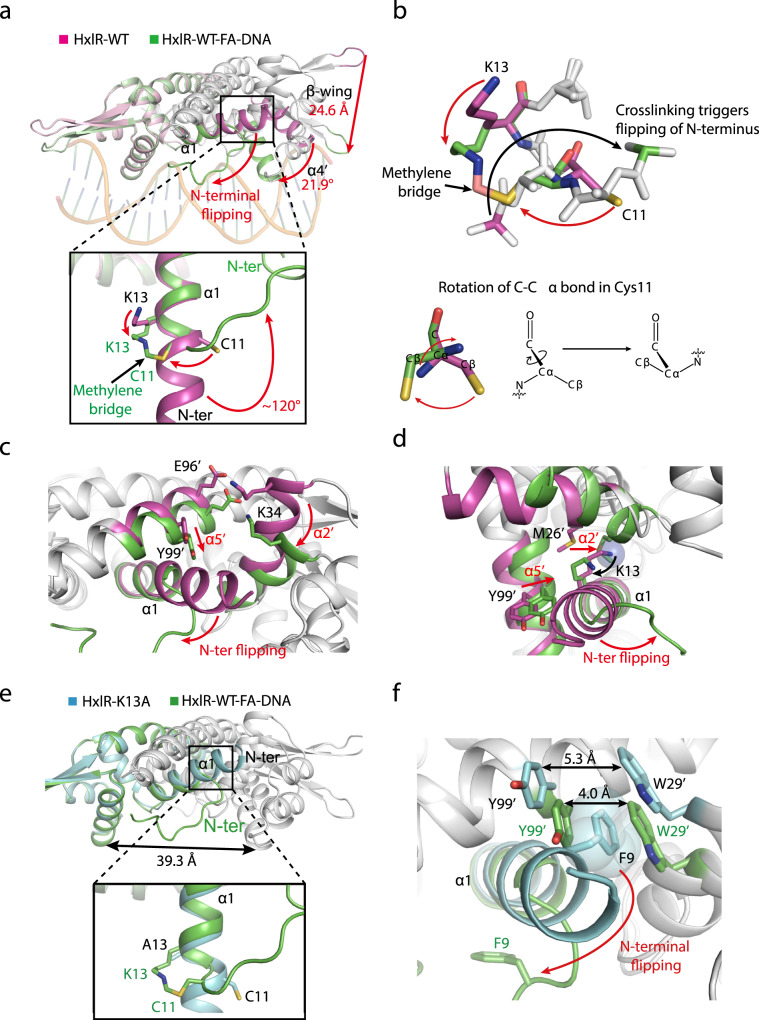
Structural bases for FA-induced HxlR activation. **a** The “N-terminal helix-flipping” mechanism of HxlR protein upon FA treatment. Superposition of HxlR-WT (magenta) and HxlR-WT-FA-DNA (green) indicates that FA induces a dramatic rearrangement on helix α1. The FA-triggered Cys11-Lys13 intrahelical crosslinking and methylene bridge formation forced the residues before Cys11 to flip ~120 degree away from the original orientation, and become unstructured. This “N-terminal helix-flipping” caused a large movement of the wHTH domain, with helix α4 rotated 21.9 degree and the tips of β-wings translocated 24.6 Å, producing an optimized conformation for DNA binding. Translocations of the structure domains and residues are indicated by the red arrow. **b** Detailed view of the crosslinking-induced “N-terminal helix-flipping”. In order to form the crosslink, C-Cα bond in Cys11 rotated to allow the sidechain thiol group to get close to Lys13 (red arrow), leading the N atom of Cys11 and the connected N-terminal residues to flip to the opposite side (black arrow). **c** The “N-terminal helix-flipping” triggered the reorganization of helices α1, α2’, and α5’, with the residues before Cys11 becoming unstructured, helix α5’ moving towards helix α1 and the concurrent rotation of helix α2’. Translocations of these helices are indicated by red arrows. **d** The FA-induced Cys11-Lys13 intrahelical crosslinking brings helix α2’ towards helices α1. The resulting methylene bridge forced helix α2’ to move closer to helix α1, with Met26’ filling the space left by Cys11-Lys13 crosslinking (shown in sphere). Red arrows indicate the movement of Lys13 and helix α2’. **e** Superposition of HxlR-K13A (cyan) and HxlR-WT-FA-DNA (green) indicates the absence of “N-terminal helix-flipping” in HxlR-K13A. Similar to that of HxlR-WT, helix α1 in HxlR-K13A extends beyond Cys11 to the N-terminal residues, with Cys11 and Ala13 located on the opposite sides. Distances between the DNA-binding helices α4 and α4′ are indicated by black arrow. Residues from HxlR-K13A and HxlR-WT are marked in black and green, respectively. **f** “N-terminal helix-flipping” is crucial for HxlR to undertake the DNA-binding conformation. As the “N-terminal helix-flipping” does not occur in the HxlR-K13A mutant, the sidechain of Phe9 is located between that of Trp29’ and Tyr99’, which causes the distance between Trp29’ and Tyr99’ larger than that of HxlR-WT-FA-DNA and prevents further approaching of HxlR subunits. The sidechain of Phe9 is shown in sphere, and the distances between Trp29’ and Tyr99’ in different conformations are indicated by black arrows.

In order to further understand how HxlR protein is activated via the FA-triggered intrahelical crosslinking reaction, we next closely inspected the difference between the structures of HxlR-WT and HxlR-WT-FA-DNA (Supplementary Note 4, Supplementary Fig. 3a–e). When the dimeric form of HxlR-WT-FA-DNA and HxlR-WT were superimposed on one monomer, the non-superimposed subunits showed a large conformational change, with the wHTH domain rotated 21.9 degree and tips of the β-wings translocated 24.6 Å (Fig. 2a). It is noteworthy that although the superimposed subunits of HxlR-WT-FA-DNA and HxlR-WT fit close to each other (r.m.s.d. = 0.595 Å for 82 Cα atoms, Supplementary Fig. 3f), an obvious difference was observed on the N-terminal residues. Whereas the N-terminal residues formed an intact helix α1 in the HxlR-WT structure, this helix breaks at Cys11 in HxlR-WT-FA-DNA, with residues before Cys11 becoming unstructured. This helix breakage is likely due to the FA-triggered Cys11-Lys13 crosslinking reaction, which produced a covalent methylene bridge between these two residues that were originally separated by 9.6 Å and located on the opposite sides of helix α1. In order to accommodate this newly formed intrahelical methylene bridge, helix α1 was forced to break at Cys11 and rotate ~120-degree that caused the N-terminal residues to flip away from the original orientation (we termed this as “N-terminal helix-flipping”, Fig. 2a, b).

We next investigated the relationships between this local “N-terminal helix-flipping” and the global conformational change on HxlR. Several MarR/DUF24 family members have been reported to undergo signal-induced local conformational change at their dimer interface that allosterically regulates DNA binding^32,33^. Similarly, close inspections on HxlR’s dimer interface indicated that the “N-terminal helix-flipping” slightly twisted helix α5’ towards helix α1, with the sidechain of Tyr99’ now filled in the space originally occupied by helix α1 before breakage (Fig. 2c). The other helix in the dimerization domain—helix α2’ now rotate accordingly to maintain its interactions with helix α5’ (e.g., the electrostatic interactions between Lys34’ and Glu96’, Fig. 2c), which further rotate the wHTH domain. Meanwhile, it should be noted that rotation of Lys13 upon crosslinking with Cys11 also promoted the rotation of helix α2’, with the side-chain of Met26’ now filled in the space originally occupied by Lys13 (Fig. 2d). Consequently, FA-triggered intrahelical crosslinking and “N-terminal helix-flipping” induced a coordinated rotation and movement of helices at HxlR’s dimer interface, which allosterically rearranged its wHTH domains to adopt a more optimal conformation for DNA binding.

Further details of the “N-terminal helix-flipping” mechanism was revealed with structure of HxlR-K13A. The 1.7-Å-resolution HxlR-K13A structure exhibited a more optimal conformation for DNA binding than HxlR-WT but had less significant changes than that of HxlR-WT-FA-DNA (Supplementary Note 5, Supplementary Fig. 3g and Supplementary Table 1). Particularly, helix α1 does not break at Cys11 in the HxlR-K13A structure and residues Cys11 and Ala13 are located on the opposite sides of an intact helix α1 (Fig. 2e), indicating that the absence of “N-terminal helix-flipping” might be the main reason why HxlR-K13A does not undertake the conformation as HxlR-WT-FA-DNA. Further inspection at the N-terminus of HxlR-K13A revealed that the sidechain of Phe9 is located between Trp29’ and Tyr99’ on the dimer interface (Fig. 2f). In contrast, Phe9 is on the opposite side of helix α1 due to the N-terminal helix-flipping on the HxlR-WT-FA-DNA structure, which shortened the distance between the rings of Trp29’ and Tyr99’ from 5.3 Å to 4.0 Å (Fig. 2f). Therefore, the translocation of Phe9 together with the movement of helices α2’ and α5’ supported the key role of the “N-terminal helix-flipping” in enforcing HxlR’s helices α1, α2’, and α5’ closer to each other, resulting in an optimal conformation for DNA binding. In the absence of the “N-terminal helix-flipping”, HxlR-K13A can only adopt an intermediate conformation between that of HxlR-WT and HxlR-WT-FA-DNA.

Therefore, we propose the structural mechanism of FA-triggered HxlR activation as follows: (1) FA crosslinks the sidechain of Cys11 and Lys13 in HxlR protein to produce an intrahelical methylene bridge; (2) This methylene crosslink triggers the “N-terminal helix-flipping” that forces the mainchain of residues in front of Cys11 to extend away from helices α2’ and α5’; (3) The sidechain of Lys13 also moves away from helix α2’, allowing the movement of helices α1, α2’, and α5’ at the dimer interface; (4) Rotation and translocation of helices α2’ and α5’ further drives the movement of the whole wHTH domain, decreases the distance of helices α4 and α4’, and induces HxlR to undertake an optimal conformation for DNA binding.

### Mechanism-inspired design and characterization of the genetically encoded FA sensors

**Fig. 3:**
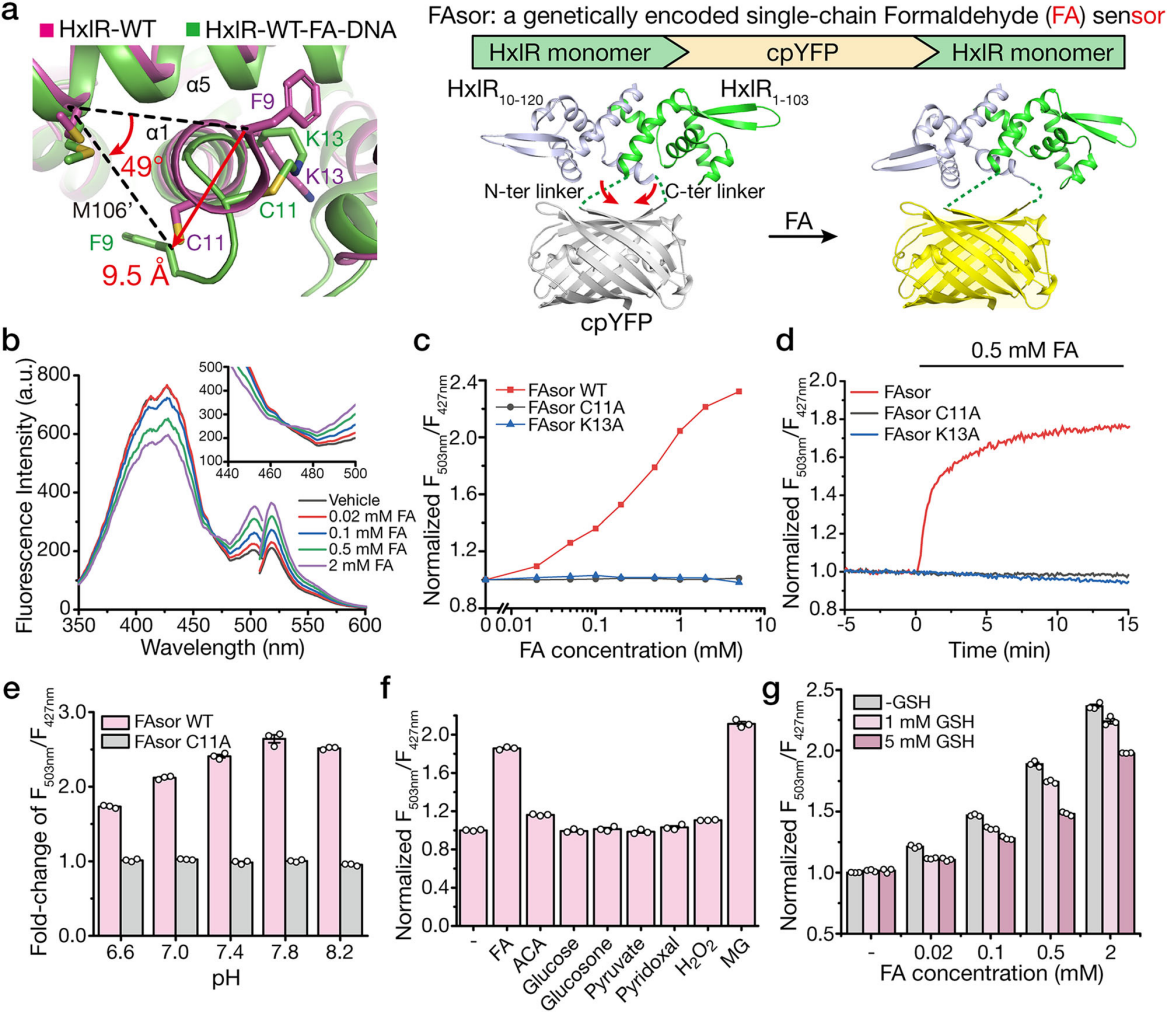
Development and characterization of the HxlR-based FA sensors (FAsors). **a** Structure-guided design of the genetically encoded FA sensor—FAsor for detecting conformational change in HxlR protein. FA-triggered Cys11-Lys13 crosslinking induces large translocation of residue Phe9 on the same helix (9.5 Å and turned 49 degree to Met106’ on helix α5). By fusion of a cpFP between two subunits in HxlR dimer protein, the resulting single-chain sensor (HxlR-cpYFP-HxlR) can transduce the FA-induced conformational change into the fluorescence signal change. Red arrows indicate distance of Phe9 translocation during conformational change. **b** Fluorescence response of FAsor in the presence of FA. Excitation and emission spectra of FAsor in the absence and presence of different concentrations of FA are shown. FAsor exhibited a ratiometric property in its excitation spectrum, with its fluorescence increased above 467 nm and decreased below 467 nm upon FA treatment. **c** The ratiometric change of FAsor in response to serial concentrations of FA ranging from 0 to 5 mM in 30 min. The ratio is calculated as the fluorescence intensity of 503-nm excitation peak divided by that of the 427-nm excitation peak, with the emission filter fixed at 516 nm. FAsor-WT responded to FA quantitatively while FAsor-C11A and FAsor-K13A mutants remained unchanged. **d** Kinetics measurement of FAsor in response to FA. 0.5 mM FA was added to 10 μM FAsor. Fluorescence ratio of FAsor-WT increase rapidly upon addition of FA, while FAsor-C11A and FAsor-K13A mutants remain stable. **e** Response of FAsor-WT/C11A to FA at different pH. The columns indicate fluorescence change of FAsor-WT/C11A before and after addition of 2 mM FA in buffers with different pH. FAsor-WT is able to sense FA between pH 6.6–8.2 with the FAsor-C11A control probe showing negligible responses in the same pH range. **f** Fluorescence response of FAsor to biologically relevant carbonyl metabolites and hydrogen peroxide. Data shown are for 0.5 mM of all species. **g** The effects of GSH on FAsor. The fluorescent response of FAsor to FA retains up to ~80% in the presence of 5 mM GSH. Data in **e**–**g** are shown in mean ± SEM for three measurements.

Inspired by this unique FA-sensing mechanism, we set out to develop a genetically encoded formaldehyde sensor by coupling the FA-induced conformational change of HxlR with local-environment-dependent fluorescence response of circularly permuted fluorescence proteins (cpFPs)^35–37^. Since the direction of Phe9 on helix α1 turned ~50 degree upon FA treatment, we chose to link two HxlR monomers through cpYFP between helices α5 and α1’ (Fig. 3a). A total of nine constructions were created and all of the resulting HxlR-cpYFP-HxlR fusion proteins (HYH) exhibited dose-dependent fluorescence responses to FA despite of different inserted sites (Supplementary Fig. 4a–l). In contrast, control experiments using cpYFP alone showed little change of fluorescence, suggesting that the fluorescence change was not caused by the perturbation of the chromophore (Supplementary Fig. 4c). Furthermore, the FA-induced conformational change happened in the absence of HxlR’s cognate DNA, which means that the optimal DNA-binding conformation of HxlR in the HxlR-WT-FA-DNA was not caused by the induced-fit effect of its cognate DNA. These results indicated that insertion of cpYFP between HxlR monomers is able to form a genetically encoded FA sensor. Encouraged by this initial attempt, we then optimized the performance by systematically varying the linker sequence between cpYFP and HxlR. Among all the variants we tested, the HYH-5 variant exhibited a ratiometric property, with an increased fluorescence response to FA when excited above 467 nm, and decreased fluorescence response below 467 nm, while all other constructions showed intensiometric decreased fluorescence response (Supplementary Fig. 4d–l). Therefore, the HYH-5 variant, which has cpYFP inserted between residues Tyr105 and Phe9’ of the two HxlR monomers and a Glu-Phe sequence spacer before the N-terminus of cpYFP, was selected and renamed as FAsor (FA sensor) for further investigations.

The ratiometric property of FAsor is highly desired for quantitative detection of analytes in living cells. We first titrated FAsor with serial concentrations of FA ranging from 0 to 5 mM. Results revealed that FAsor exhibited a dose-dependent fluorescence response to FA, with an approximately 2.2-fold enhanced signal at 2 mM, and a detectable response to FA at a concentration as low as 20 μM (Fig. 3b,c). As for negative control, we introduced the Cys11-to-Ala or Lys13-to-Ala mutation to both HxlR monomer proteins in FAsor. The resulting variants FAsor-C11A and FAsor-K13A had no response to FA up to 5 mM (Fig. 3c). These results together proved that both Cys11 and Lys13 are essential for FA-induced conformational change on HxlR protein, and ruled out the perturbance of the chromophore by environment changes or other damaging effects. Following kinetic characterization showed that the FAsor had a rapid response to FA treatment with >80% change occurred within 5 min (Fig. 3d), which is consistent with the time-scale for HxlR-regulated transcriptional activation in vivo^38^. Consistent with the essential roles of Cys11 and Lys13 for FA sensing, both FAsor-C11A and FAsor-K13A showed negligible change after addition of 0.5 mM FA during the entire measurement (Fig. 3d). Considering the sensitivity of cpYFP to pH^35^, we next investigated the effects of buffer pH on fluorescence properties. FA is able to induce fluorescence change of FAsor-WT in buffers with pH between 6.6 and 8.2, showing that the ratiometric change of FAsor can reliably detect FA within the pH range (Fig. 3e). In contrast, the *F*_503nm_/*F*_427nm_ ratio of FAsor-C11A did not respond to FA in the same pH range (6.6–8.2; Fig. 3e), indicating this variant can thus be utilized as a control probe to rule out the effect of pH change. The specificity of FAsor was also verified with various carbonyl-containing small molecule metabolites including acetaldehyde (ACA), methylglyoxal (MG), glucose, glucosone, pyruvate, pyridoxal as well as hydrogen peroxide. Most of the tested compounds caused negligible changes of FAsor’s fluorescence except for MG, which induced similar response with FA (Fig. 3f). The result was not unprecedented as HxlR has been shown to respond to methylglyoxal in *B. subtilis*^38^. Yet we consider it has limited effect on in vivo application of FAsor in most cases, as MG has a much lower endogenous concentration than FA^39^. Notably, despite exhibiting a 20% decrease of the fluorescence response, FAsor can still sense FA in the presence of 5 mM glutathione (GSH) (Fig. 3g). Our results indicated that FAsor can preferentially form the covalent crosslinking with FA under this condition, allowing detection of FA in the presence of physiological concentrations of glutathione.

### Subcellular visualization of FA by FAsors in living cells

**Fig. 4:**
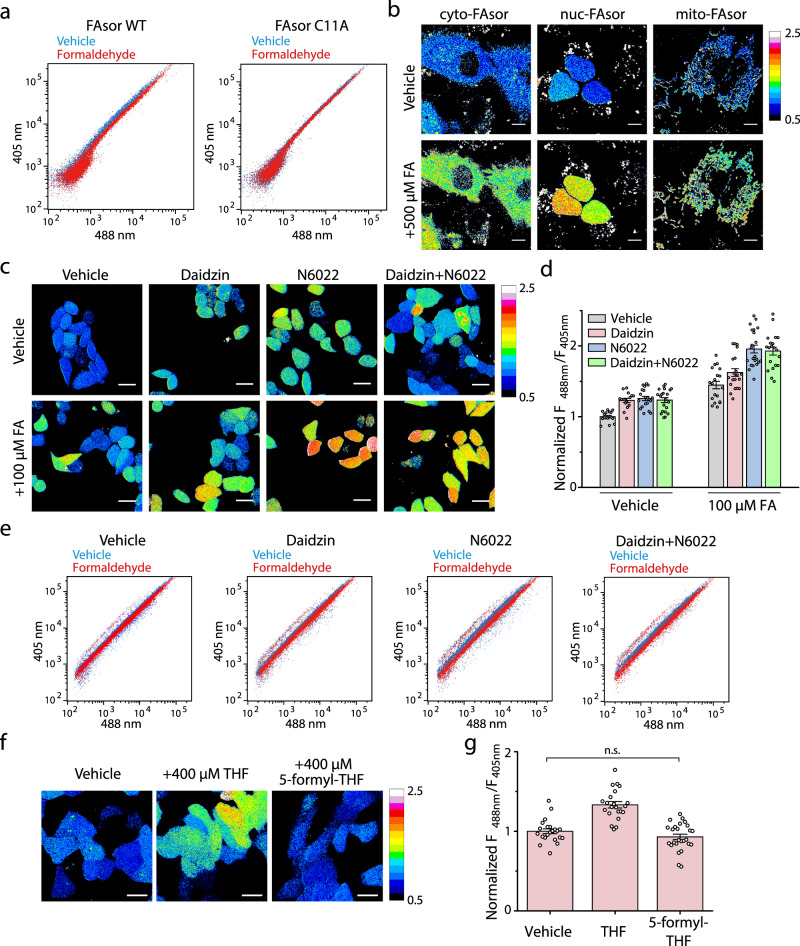
Spatial and temporal visualization of FA by FAsors in living cells. **a** FACS measurements of FAsor’s *F*_488nm_/*F*_405nm_ ratio in response to exogenous FA in HEK293T cells. Cells are incubated at 37 °C with 0.5 mM FA for 1 h. FA induced the increase of *F*_488nm_/*F*_405nm_ ratio for cells expressing FAsor-WT but not cells expressing FAsor-C11A. **b** Fluorescence imaging of subcellular-targeted FAsor upon the treatment of FA. HeLa cells were treated with 0.5 mM FA for 30 min. FA levels in cytosol, nucleus or mitochondria all undergo changes in response to exogenously added FA. Scale bars, 10 μm. **c**, **d** Fluorescence imaging of FAsor without subcellular-targeting sequence in response to endogenous and exogenous FA in different states of FA metabolism. HeLa cells were treated with or without the inhibitors daidzin and/or N6022 for FA-degrading enzymes for 3 h, followed with incubation with or without 0.1 mM FA for 1 h. The enhanced *F*_488nm_/*F*_405nm_ ratio on inhibitor-treated cells indicates higher intracellular FA level due to the suppressed FA degradation. **d** is the group summary of *F*_488nm_/*F*_405nm_ under different conditions. For groups from left to right, *n* = 16, 15, 20, 21, 19, 20, 22, and 20 cells, respectively. Scale bars, 25 μm. **e** FACS measurements of FAsor’s *F*_488nm_/*F*_405nm_ ratio in response to FA in different states of FA metabolism. HEK293T cells are incubated with different inhibitors (10 μM each) at 37 °C for 3 h, followed with or without addition of 0.4 mM FA for another 1 h. **f**, **g** Fluorescence imaging of the FA generation via THF metabolism by FAsor without subcellular-targeting sequence. HeLa cells were treated with 0.4 mM THF or 5-formyl-THF for 30 min. The increased *F*_488nm_/*F*_405nm_ ratio indicated endogenously generated FA molecules. **g** is the group summary of *F*_488nm_/*F*_405nm_ under different conditions. For groups from left to right, *n* = 23, 22, and 26 cells, respectively. Unpaired two-sided student’s *t-*test was performed. n.s. no significance (*P* = 0.12). Scale bars, 20 μm. In **b**, **c**, **f**, images were pseudocolored with normalized *F*_488nm_/*F*_405nm_ ratio. Data in **d**, **g** are shown in mean ± SEM.

Having established that FAsors can respond to FA in vitro, we next evaluated their ability to sense FA in living cells. Flow cytometry showed an increase of *F*_488nm_/*F*_405nm_ ratio of cells transfected with FAsor-WT upon treatment of exogenous FA (Fig. 4a, Supplementary Fig. 5a). In contrast, cells transfected with FAsor-C11A showed negligible response to FA, indicating that the fluorescence change was induced by FA-induced conformational change of HxlR but not other changes in cellular environment (Fig. 4a). We next utilized FAsor to visualize subcellular change of FA by confocal microscopy. Different subcellular-targeting sequence, including nucleus, cytoplasm and mitochondria, were fused to FAsor to generate subcellular-targeting FAsor variants, which all showed the desired subcellular localizations (Supplementary Fig. 5b). Imaging with nuclear-, cytosolic- and mitochondrial-FAsor in HeLa cells all showed the increased *F*_488nm_/*F*_405nm_ ratiometric change upon FA treatment (Fig. 4b, Supplementary Fig. 5c). As controls, the subcellular-targeting variants of the FAsor-C11A showed no obvious change upon FA (Supplementary Fig. 5d, e). Furthermore, we also simultaneously visualized FA levels in nucleus and cytosol with FAsor variants that have different colors of cpFPs inserted (Supplementary Note 6, Supplementary Fig. 5f, g). Together, these results indicated that the genetically encoded, subcellular-targeted FAsors offered a convenient toolkit for dynamic monitoring of FA levels in different subcellular compartments within living cells.

### Visualization of endogenous FA dynamics in living cells by FAsors

Then we turned our attention to investigate the dynamic production of endogenous FA inside cells. FA is continuously produced and consumed and the homeostasis of FA is dynamically regulated inside cells. Since our in vitro measurements indicated that GSH is able to compete with FAsor on reaction with FA, we first investigated the effect of GSH on FAsor signal in cells. We first incubated cells with and without FA, followed by changing to medium with addition of mono-ethyl-GSH, which is membrane-soluble and able to release GSH inside cells. After addition of mono-ethyl-GSH, the FAsor signal dropped to lower than the basal level (Supplementary Fig. 5h, i). These results indicated that GSH is able to reverse the reaction of FAsor and FA. Recent works indicated that interruption of ALDH2 or ADH5, the major enzymes for FA degradation, may cause the accumulation of DNA damage and related diseases such as bone marrow failure, as well as dysfunction of liver and kidney^4–6^. We utilized inhibitors to simulate the ALDH2/ADH5 deficient condition to investigate the endogenous FA fluctuations^40,41^. When the FAsor-transfected HeLa cells were treated with daidzin (ALDH2 inhibitor), N6022 (ADH5 inhibitor) alone or together, and vehicle control respectively, cells with different treatment of inhibitors all showed increased *F*_488nm_/*F*_405nm_ ratio than that of vehicle-treated cells (Fig. 4c, d). Moreover, all of inhibitors-treated cells showed higher response upon subsequent incubation with 0.1 mM FA (Fig. 4c, d). The higher response upon subsequent incubation of FA had also been verified by flow cytometry (Fig. 4e, Supplementary Fig. 6a). This result confirmed that the deficiency of FA metabolism in ALDH2/ADH5 inhibited cells disrupted FA homeostasis and caused cellular FA accumulation, which may lead to cell damaging effects in related diseases. In addition, a recent report showed that inhibition of GSH synthesis with L-BSO (L-Buthionine-Sulfoximine, an inhibitor of γ-glutamylcysteine synthetase) promoted the toxicity of FA to cells, especially in those with ADH5 knockout^42^. We thus treated HeLa cells with L-BSO and N6022 alone or together, and vehicle control, respectively. L-BSO induced similar FAsor signal increase compared with N6022 (Supplementary Fig. 6b, c). Combined treatment of L-BSO and N6022 resulted in little increase with no statistical difference (Supplementary Fig. 6b, c), indicating that ADH5 and GSH synthesis might be epistatic for the control of intracellular FA^42^.

Next, we employed FAsor to visualize endogenous FA dynamics in tetrahydrofolate (THF) metabolism. A recent work indicated that oxidative THF decomposition can release FA, which may serve as the source of 1C units and support nucleotide synthesis^12^. We thus utilized FAsor to detect endogenous FA fluctuation upon the addition of THF. A significant increase of *F*_488nm_/*F*_405nm_ ratio was observed on HeLa cells treated with 0.4 mM THF (Fig. 4f, g). In contrast, treatment of cells with 5-formyl-THF, which is resistant to oxidation and unable to release FA^12^, induced negligible signal changes (Fig. 4f, g). In summary, we have established that FAsors could detect endogenous FA fluctuation and metabolism.

### Visualization of FA dynamics in mouse brain tissues by FAsors

**Fig. 5:**
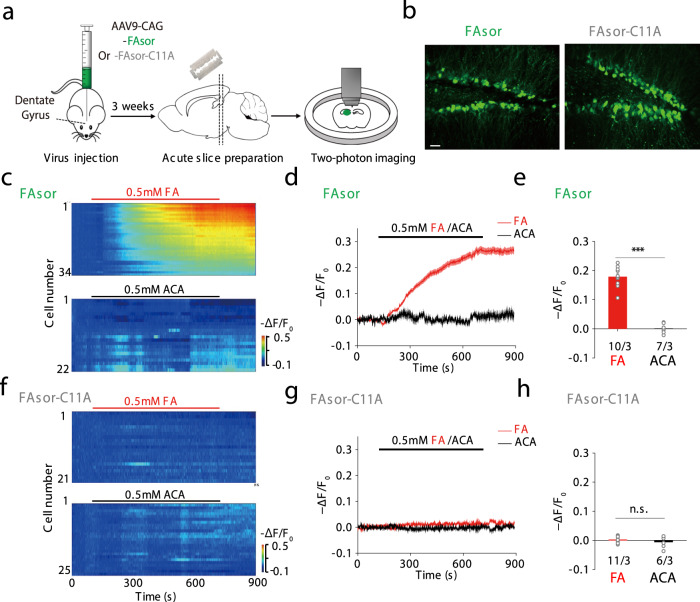
Visualization of FA in mouse acute brain slices by two-photon imaging of FAsors. **a** Schematic illustration of the workflow. AAVs containing FAsor or FAsor-C11A were injected into the dentate gyrus of mice, and acute brain slices were prepared 3 weeks after viral expression, which were placed under two-photon microscope for imaging. **b** Expression of FAsor and FAsor-C11A in pyramidal neurons of the dentate gyrus in acute brain slices. The two-photon laser was set at 880 nm for excitation. Scale bars: 20 μm. **c**–**e** The fluorescence responses of FAsor-expressing neurons to exogenous perfusion of 0.5 mM formaldehyde (FA) (upper, red), or 0.5 mM acetaldehyde (ACA, lower, black) as a control. The responses of individual cells in one single slice were plotted in pseudo-color in **c**, and their averaged time-dependent traces were shown in **d**. The group data of responses in multiple slices of different animals were summarized in **e** with data shown in mean ± SEM. Unpaired two-sided student’s *t-*test was performed. *n* = 10 slices from 3 mice and 7 slices from 3 mice, for FA and ACA, respectively. ***P < 0.001 (*P* = 3.19 × 10^−7^). **f**–**h** Similar as **c**–**e**, except the fluorescence responses of FAsor-C11A-expressing neurons were summarized and plotted. Data are shown in mean ± SEM in **h**. Unpaired two-sided student’s *t*-test was performed. *n* = 11 slices from 3 mice and 6 slices from 3 mice, for FA and ACA, respectively. n.s. no significance (*P* = 0.35).

Compared with small molecule probes, genetically encoded fluorescent sensors provide the possibility of cell-type-specific expression as well as potential long-term imaging in animals. Cells transfected with FAsor or infected with Adeno-associated virus (AAV) containing FAsor could respond to FA with fluorescence changes under confocal or two-photon microscope, while cells expressing FAsor-C11A did not respond to FA (Supplementary Fig. 7a–d). To test the applicability of FAsor in detecting FA dynamics in a physiological relevant system, we injected AAV containing either FAsor or FAsor-C11A in the hippocampus dentate gyrus region of mice, and prepared acute brain slices 3 weeks after in vivo viral expression (Fig. 5a). Both FAsor and FAsor-C11A showed robust fluorescent signal in the pyramidal neurons of the dentate gyrus under two-photon microscope (Fig. 5b), indicating their successful expression.

Next, we treated the sensor-expressing acute brain slices with exogenous perfusion of FA or acetaldehyde (ACA). The application of 0.5 mM FA elicited fluorescence signal decrease in FAsor-expressing neurons at single cell level, with the averaged ∆F/F around −30% (Fig. 5c–e, red), while ACA did not cause detectable fluorescence changes (Fig. 5c–e, black), ruling out the possibility of fluorescence change caused by environmental changes. Furthermore, in neurons expressing FAsor-C11A, application of either ACA or FA did not cause observable fluorescence changes (Fig. 5f–h), consistent with the result at protein level (Fig. 3c). Altogether, these results indicated that FAsor could specifically detect the dynamics of FA in mouse brain tissues.

## Discussion

FA has long been considered as a detrimental molecule, with the potential physiological role remaining controversial^1,5,6,12^. This is largely due to the lack of direct evidence supporting the beneficial effects of FA on a given biomolecule or cellular machinery as opposed to the nonspecific damaging effects. Our study on the FA-sensing transcription factor HxlR revealed that FA induced a specific intrahelical crosslinking reaction between residues Cys11 and Lys13. The resulting methylene bridge (Cys-CH_2_-Lys) on HxlR’s helix α1 caused unwinding and rotation of its N-terminus (“N-terminal helix-flipping”) that allosterically rotated its DNA-binding domain with enhanced DNA affinity and transcriptional activation. This FA-triggered covalent Cys-Lys crosslinking represents a modification on proteins with a gain-of-function effect. This may shed light on how cells manage to sense highly reactive species such as FA. Furthermore, in addition to FA, other RCS with multivalent reactive groups may also influence protein conformation and functions through intramolecular or intermolecular crosslinking. All these findings represent a general mechanism how metabolic pathways interact with stress-responsive signaling through electrophiles, especially in a gain-of-function crosslinking manner^43–46^, which merits further investigation by quantitative proteomics in the future.

Recent works have revealed a RcnR/CsoR family derepressor, *E. coli* FrmR, that responded to FA via crosslinking between cysteine and the N-terminal proline^29^. The secondary amine group from the proline residue is essential, as mutating it to primary amine (e.g., Pro to Ser/Ala mutation) abolished FA sensing by FrmR in vivo^29,30^. In contrast, HxlR represented an example in which protein may also utilize the side-chain of primary amine for FA sensing. The reason why HxlR can utilize primary amine (sidechain of Lys13) to sense FA is yet to be investigated. Despite that the FA-reacting residues are different, the similar crosslinking reactions between the thiol group and amine in HxlR and FrmR exhibited how FA regulatory proteins specifically distinguish this signal from other thiol-reactive species such as ROS. Another interesting question is whether the FA-induced crosslinking on regulator proteins, e.g., HxlR/FrmR, is reversible. Recent works indicated that timonacic, the product of cysteine crosslinked by FA, was able to slowly convert back to formaldehyde and cysteine^47^. The authors incubated ^12^C-timonacic with ^13^C-FA in PBS solution (pH 7.4) and found the sample switched to almost 100% ^13^C-timonacic after 12 h^47^. As FA-crosslinking resulted a five-member-ring in timonacic, which is highly stable and not existed in the proteins, we hypothesized that the FA-induced crosslinking on regulator proteins can be reversed in a faster kinetics. The intracellular thiols may further promote the reverse reaction of FA-induced crosslinking. Additionally, the degradation of the FA-crosslinked proteins in cells, such as through the Clp-dependent proteolysis, is also a potential mechanism in physiological environment.

Inspired by the unique FA-sensing mechanism of HxlR, we developed genetically encoded sensors-FAsor and FAsorRed that were able to transduce the FA-induced protein conformational change to a fluorescent signal. This allowed us to detect and visualize FA dynamics in various physiological contexts with high spatial-temporal resolution. Recently, a number of groups made developments of small molecule FA sensors, which can be classified as 2-aza-Cope-based sensors and formimine/aminal-based sensors by their sensing mechanism^21,22^. 2-aza-Cope-based sensors have high sensitivity and specificity for FA, but with low reaction kinetics (may take hours to get reasonable signal change) and are irreversible^17,18^. Formimine- and aminal-based sensors usually react rapidly and reversible with FA (the signal changes within 10 sec to a few minutes), while they also respond to simple aldehydes such as acetaldehyde^19,20^. The reaction kinetics, sensitivity and specificity of FAsor, which can sense 20 μM FA within 5 min, are more similar with the formimine/aminal-based small molecular sensors. As a ratiometric sensor, FAsor have lower turn-on ratio compared to the most intensiometric small molecular FA sensors (some of which may have 900-fold turn-on upon reaction with FA). Instead, FAsor avoided from suffering the signal fluctuation induced by varied quantity of the sensor. FAsor has the dual specificity towards FA and MG as HxlR is able to respond to both molecules. As MG has a much lower endogenous concentration than FA^39^, in most cases the main signal change of FAsor in vivo would be induced by FA. The application of FAsor would be most appropriate when the FA source is clear, or distinguishing signals from FA and MG is not crucial, such as the preliminary screening whether FA/MG level were enhanced in a disease model or under certain treatment, specific process. Further research on sensing mechanism of HxlR to MG may lead to the development of MG and FA specific sensors. Notably, another advantage for the genetically encoded, fluorescent protein (FP)-based sensors is subcellular targeting. Subcellular-targeted FAsor variants were also developed for simultaneously monitoring FA in different subcellular compartments. Our sensors provide a toolkit that may help to improve our understanding of the potential roles of FA in various cellular processes. Some recent research examined Fanconi anemia patients and found that people holding the ALDH2 dominant-negative variant (ALDH2*2) had the tendency of accelerated progression of bone marrow failure (BMF)^48,49^. Study on *Fancd2*, *Aldh2* and *Adh5* knockout mice have indicated the effect of increased endogenous formaldehyde in the process^4,5^. We have confirmed elevated endogenous formaldehyde upon inhibition of ALDH2 with FAsor, and the putative role of endogenous formaldehyde in ALDH2*2 alleles worth further investigation. In particular, the application of FAsors in mouse brain slices may facilitate the study of the underlying connections between FA and neurodegenerative diseases.

## Methods

### Primary cultures

Rat cortical neurons were prepared from postnatal 0-day old (P0) Sprague–Dawley rat pups (male and female, random choice; Beijing Vital River). The cortical neurons were dissociated from the dissected rat brains in 0.25% Trypsin-EDTA (GIBCO), and plated into LabTek 8-well chambered coverglass (NUNC) in neurobasal medium (GIBCO) containing 2% B-27 supplement (GIBCO), 1% GlutaMax (GIBCO), and 1% penicillin-streptomycin (GIBCO). The neurons were cultured at 37 °C in 5% CO_2_.

### Bacterial strains

*E. coli* strains DH5α and BL21(DE3) were purchased from Tiangen Biotech. *Bacillus subtilis* strains 168 was purchased from American Type Culture Collection (ATCC). *E. coli* strains BW25113 was obtained from the National BioResource Project (National Institute of Genetics, Japan).

### Cell lines

HEK293T cell line (female, ATCC, CRL-11268) and HeLa cell line (female, ATCC, CCL-2) were purchased from ATCC. The cell lines were authenticated by the morphology under microscopy and the growth curve analysis. Cells were maintained in DMEM (GIBCO) media at 37 °C in 5% CO_2_. All cell lines were supplemented with 10% fetal bovine serum and 1% penicillin-streptomycin (GIBCO).

### Mice

Postnatal 56- to 70-day-old (P56-70) wild-type C57BL/6 mice were used to prepare the acute brain slices (male and female, random choice; Beijing Vital River). All mice were either family-housed or pair-housed in a temperature-controlled room (21.5 degree centigrade) with a 12-h/12-h light/dark cycle, with humidity controlled as 55%. All procedures for animal surgery and maintenance were performed using protocols that were approved by the Animal Care & Use Committees at Peking University, and were performed in accordance with the guidelines established by US National Institutes of Health guidelines.

### Plasmids construction

The gene of HxlR (UniProt number: P42406 [https://www.uniprot.org/uniprot/P42406]) from strains 168 of *Bacillus subtilis* was subcloned into pET28a between sites NdeI and XhoI with stop codon before C-terminal his-tag. Site-directed mutagenesis was applied to obtain HxlR mutants based on the QuickChange™ method developed by Stratagene (La Jolla, CA). Oligonucleotides used for the mutagenesis reactions are listed in Supplementary Table 2. The *hxlAB-gfp* reporter plasmid was constructed by subcloning *hxlAB* promoter from *B. subtilis* 168 into pL(marO)-GFP plasmid between sites XhoI and KpnI, replacing the original marO promoter^50^. pBAD-HxlR was constructed by replacing the DiZPK-RS and Mb-tRNA-pyl in pSupAR plasmid with the gene of HxlR protein via Gibson Assembly^51,52^. Constructions of HxlR-cpYFP-HxlR-expressing plasmids were achieved via Gibson Assembly and/or QuickChange™ method^51^. For eukaryotic expressing plasmids (pcDNA-based plasmids), a synthesized codon-optimized HxlR gene (by Genewiz, the sequence is listed in Supplementary Table 3) was utilized as template.

### Protein expression and purification

For expression of HxlR protein, pET28a-HxlR was transformed into *E. coli* BL21(DE3) strain. Then a single colony was collected and cultured overnight, followed by diluting into 1 L LB medium with the ratio of 1:100. For expression of HxlR mutant, the culture was incubated in a shaker at 37 °C for 3 h (to OD_600nm_ ~0.6), and added with 1 mM IPTG (isopropyl β-D-1-thiogalactopyranoside). The shaker temperature was then adjusted to 30 °C and the bacteria were harvested after 5 h incubation. For expression of FAsor, FAsorRed and other HxlR-cpYFP-HxlR protein, the bacteria carrying expressing plasmids based on pET28a was incubated at 25 °C for 8 h after addition of IPTG.

Cell pellets were resuspended in lysis buffer (20 mM HEPES pH = 7.5, 300 mM NaCl, 10% glycerol). After sonicating on ice for 20 min, lysate of bacteria was centrifuged and the supernatant was loaded onto a 5-ml HisTrap HP column (GE Healthcare) before the linear gradient procedure being applied to elute the protein (buffer A: 20 mM HEPES pH = 7.5, 300 mM NaCl, 10% glycerol; buffer B: 20 mM HEPES pH = 7.5, 300 mM NaCl, 500 mM Imidazole, 10% glycerol). The HxlR components were collected and further concentrated. Overnight thrombin digestion (HxlR protein diluted to 1 mg/ml in 20 mM Tris-HCl, pH = 8.4, 150 mM NaCl, 2.5 mM CaCl_2_ with 1 U/ml thrombin added) at 16 °C was performed to remove the N-terminal His-tag. After digestion, the protein sample was treated with 10 mM DTT for 15 min to reduce potential oxidation and next purified by size exclusion chromatography with HiLoad 16/60 Superdex 200 (GE Healthcare) equilibrated with 20 mM HEPES pH = 7.5, 300 mM NaCl. All the pH’s of the buffers were measured at room temperature. The purifications were performed at 4 °C.

### Crystallization, X-ray data collection, and structure determination

All the crystallization experiments in this study were performed with sitting-drop vapor diffusion at 20 °C. Crystals of HxlR-WT and HxlR-K13A were screened with kits from Hampton Research (Index, SaltRx, Crystal Screen, PEGRx, PEG/Ion Screen). 1.5 μl HxlR protein solution (~5 mg/mL in 20 mM HEPES pH = 7.5, 300 mM NaCl) and 1.5 μl reservoir solution were mixed. Single crystals could be observed after 24 h. For crystallization of HxlR-WT-FA-DNA complex, HxlR-WT (~5 mg/mL in 20 mM HEPES pH = 7.5, 150 mM NaCl) was treated with 1 mM FA for 1 h at 37 °C. This solution was next mixed with the same volume of double-stranded DNA solution (final Protein:DNA ratio = 1:1.5 or 1:2) for at least 30 min at 4 °C. The final mixtures were screened with reservoir solutions containing 0–100 mM NaCl, 50–200 mM MgCl_2_, 100 mM buffering reagent (Bis-tris, MES or sodium cacodylate) at various pH (5.0–7.0), and different concentrations of PEGs (PEG 1000, 2000, 3350, 4000, 6000, 8000, PEG MME 550, 5000) (w/v or v/v 10–40%). The sequence of DNA generating crystals of HxlR-WT-FA-DNA complex that has the best diffraction was 5’- CAG TAT CCT CGA GGA TAC TG -3’. The growing conditions for crystals with the best diffraction quality are 0.1 M sodium acetate trihydrate pH = 4.6, 1.5 M ammonium chloride, 10 mM DTT for HxlR-WT, 0.2 M DL-Malic acid pH 7.0, 20% w/v PEG 3350 for HxlR-K13A and 0.1 MES pH = 6.4, 50 mM MgCl_2_, 25% v/v PEG MME 550 for HxlR-WT-FA-DNA.

Crystals were washed with cryoprotectant freshly made by mixing reservoir solution with 90% glycerol in 7:2 ratio, and then flash frozen in liquid nitrogen prior to X-ray diffraction. Diffraction datasets of HxlR-WT and HxlR-WT-FA-DNA were collected by beamlines of BL17U1 at Shanghai Synchrotron Radiation Facility (SSRF)^53^ with wavelength at 0.9792 Å for HxlR-WT and 0.9793 Å for HxlR-WT-FA-DNA, respectively. Diffraction datasets of HxlR-K13A were collected by beamlines of BL19U1 at SSRF with wavelength at 0.9785 Å. Diffraction datasets were collected with Blu-Ice software. The collected datasets were processed with HKL2000 or HKL3000^54,55^, and structures were solved via molecular replacement with programs Phaser^56^ in CCP4 package^57^. Structures of HypR (Protein Data Bank code 4A5N [https://www.rcsb.org/structure/4A5N])^32^ and QsrR-DNA complex (Protein Data Bank code 4HQE [https://www.rcsb.org/structure/4HQE])^33^ are used as search models for HxlR protein and HxlR-WT-FA-DNA complex, respectively. Refinement was performed by programs REFMAC5^58^ in CCP4 package^57^, Phenix^59^ and COOT^60^. The Ramachandran plot given by MolProbity^61^ in Phenix^59^ showed that in every structure, no residue was in the disallowed region. Protein sequence alignment was performed by ClustalO^62^. PyMOL was used to generate all the figures. Summary of the data statistics is listed in Supplementary Table 1.

### Mass spectrometry and data analysis

For FT-MS analysis, purified HxlR (at 100 μM) was incubated with 1 mM FA in 20 mM HEPES pH = 7.5, 300 mM NaCl for 1 h at 37 °C. The protein sample was next desalted by Bio-Rad Micro Bio-Spin 6 column and subjected to mass spectrometric analysis by Bruker Solarix XR system. For MS-MS analysis of HxlR protein, 0.6 mM FA was added to the medium of *E. coli* BL21(DE3) expressing HxlR at 1 h before harvest. The FA-treated cells were then collected and HxlR protein was purified with HisTrap HP column as described above. The purified protein was then digested with trypsin and the resulted peptide sample was next desalted with Thermo Scientific Pierce C18 tips and subjected to Thermo Scientific Q Exactive Plus Orbitrap LC-MS/MS system, with the data collected by Xcalibur software. For LC-MS analysis of synthetic 7-amino acid peptides, the peptides were incubated with 1 mM FA in water for 1 h at 37 °C. The peptide samples were then analyzed with a Waters ACQUITY UPLC I-Class SQD 2 MS spectrometer with electrospray ionization (ESI). Data collection was achieved by Masslynx software.

### Electrophoretic mobility shift assays (EMSA)

EMSA was performed as described previously^50^. Briefly, 100 μM HxlR proteins (HxlR-WT, HxlR-C11A and HxlR-K13A) were incubated with or without 1 mM FA in 20 mM HEPES pH = 7.5, 300 mM NaCl for 1–2 h at 37 °C. After incubation, the proteins were diluted to a final volume of 20 μl containing 50 nM annealed double-stranded DNA in binding buffer (20 mM HEPES pH 7.0, 50 mM KCl, 5 mM MgCl_2_, and 10% glycerol). Sequences of oligonucleotides used for the annealing process was listed in Supplementary Table 2.

### Flow cytometry analysis of HxlR-mediated transcription *E. coli*

An overnight *E. coli* BW25113 culture harboring both the *hxlAB-gfp* reporter plasmid and the pBAD-HxlR plasmid was inoculated (1:100) in LB medium and grown to an OD_600nm_ of 0.6. L-Arabinose with 4 mM (~0.06% w/v) in final concentration was added to induce expression of HxlR protein. After 1 h induction, bacterial cells were next treated with or without 600 μM FA, 40 min before being analyzed by flow cytometry. Fluorescence of the GFP channel (EX 488 nm, EM 530/30BP) were measured with the BD LSRFortessa flow cytometer (BD Biosciences). To assess the effect of FA on viability of bacteria, *E. coli* BW25113 grown to an OD_600nm_ of 0.6 were allow to grow at 37 °C for 1 h, and followed by treating with/without 600 μM FA for 40 min. Next, 10 folds of serial dilutions of the bacteria with/without FA-treatment analysis were plated for colony counts.

### Expression of HYH-5 in cultured cells

HEK293T cells were cultured in 24-well culture plates (Corning) and HeLa cells were cultured in LabTek 8-well chambered coverglass (NUNC), and grown to a confluency of ~50% for transfection. Lipofectamine 2000 (Invitrogen) were used for transfection of HEK293T cells and XTremeGene HP (Roche) for transfection of HeLa cells. Transfection was performed according to the manufactures’ protocols. Imaging was performed 18–24 h after transfection.

The cultured neurons were infected ~10 days later after dissection using AAV. The infection was performed by adding 1 μL AAV9-CAG-FAsor/FAsor-C11A into the medium in one well of 24-well plate. Imaging was performed 48–72 h after infection.

### In vitro measurement of fluorescence

Generally, 10 μM purified FAsor/FAsorRed protein was incubated with different concentrations of FA or other compounds for 5 min before recording the fluorescent signal. All of the experiments were performed in 20 mM HEPES pH = 7.5, 300 mM NaCl at room temperature on the Cary Eclipse Fluorescence Spectrophotometer (Agilent Technologies, U.S.A.). For excitation spectra of FAsor/FAsorRed protein, intensity of fluorescence emission at 516 nm or 594 nm was measured respectively if not specified. And for emission spectra of FAsor/FAsorRed protein, the excitation filter is fixed at 503 nm or 569 nm, respectively. In the kinetics study, fluorescence emission intensities at 516 nm of FAsor excited at 427 nm and 503 nm were recorded every 6 s, respectively. Cary Eclipse Software was used for data collection.

### Flow cytometry analysis of cultured cells

For flow cytometry, HEK293T cells transfected with FAsor in a 24-well culture plate (Corning) were changed to fresh DMEM with different reagents. After incubation the cells were trypsinized and resuspended into 0.5 mL PBS. Fluorescence of the AmCyan channel (EX 405 nm, EM 525/50BP after 505LP) and FITC channel (EX 488 nm, EM 530/30BP) were measured with the BD LSRFortessa flow cytometer (BD Biosciences).

### Fluorescence imaging in cultured cells

Chambers with HeLa cells were changed to fresh DMEM without phenol red, and subjected to confocal imaging with a LSM700 laser scanning confocal microscopy (ZEISS). Cells expressing FAsor were imaged with both the 405 nm and 488 nm excitation, while cells expressing FAsorRed were imaged with the 555 nm excitation. Live cell time lapse imaging was performed in an incubator maintained at 37 °C with 5% CO_2_. For imaging of subcellular-targeting FAsor constructs, Hoechst33342 or MitoTracker DeepRed (ThermoFisher) was stained before imaging according to the manufactures’ protocols. Imaging files were collected via ZEN software and processed with ImageJ 1.52a (NIH) to measure fluorescence intensity and to generate the ratiometric pseudo-color images.

### Fluorescence imaging in brain slices

Brain slice imaging was performed as described previously^25^. In brief, the animals were anesthetized with Avertin, and acute brain slices containing the hippocampus region were prepared in cold slicing buffer containing: 110 mM choline-Cl, 2.5 mM KCl, 1.25 mM NaH_2_PO_4_, 25 mM NaHCO_3_, 7 mM MgCl_2_, 25 mM glucose, and 2 mM CaCl_2_. Slices were allowed to recover at 35 °C in oxygenated Ringers solution containing: 125 mM NaCl, 2.5 mM KCl, 1.25 mM NaH_2_PO_4_, 25 mM NaHCO_3_, 1.3 mM MgCl_2_, 25 mM glucose, and 2 mM CaCl_2_ for at least 40 min before experiments. An Olympus FV1000MPE two-photon microscope equipped with a ×40/0.80 NA water-immersion objective and a mode-locked Mai Tai Ti:Sapphire laser (Spectra-Physics) were used for imaging the slices. The two-photon excitation wavelength was set as 880 nm, except where indicated otherwise. Formaldehyde or acetaldehyde were added to the physiological solution and perfused into the chamber with slices during imaging. Imaging files were processed with ImageJ 1.52a (NIH) to measure fluorescence intensity and to generate the ratiometric pseudo-color images. Intensity traces were generated by Origin 2018 and pseudo-color heatmap images were generated by custom-written MATLAB programs.

### Statistics and reproducibility

Unless otherwise indicated, data are shown as mean and standard error of mean (SEM), and error bars in figures represent SEM. Unpaired two-sided student’s *t*-test was used to determine the *P* value and no adjustments were made for multiple comparisons in all the statistical test. For EMSA and fluorescence imaging experiments, similar results were obtained from three independent experiments.

### Reporting summary

Further information on research design is available in the Nature Research Reporting Summary linked to this article.

## Supplementary information

Supplementary Information

Reporting Summary

## Data Availability

Atomic coordinates and structure factors for the crystal structures have been deposited with accession codes PDB ID 7BZD [10.2210/pdb7bzd/pdb] for HxlR-WT, 7BZE [10.2210/pdb7bze/pdb] for HxlR-K13A and 7BZG [10.2210/pdb7bzg/pdb] for HxlR-WT-FA-DNA, respectively. The PDB accession code 4A5N corresponding to the HypR protein and PDB accession code 4HQE corresponding to the QsrR-DNA complex was used in this study. The UniProt accession codes P42406 was also used in this study. Other data are available from the corresponding author upon reasonable request. Source data are provided with this paper.

## Source data

Source Data

## Code availability

All codes and programs will be provided upon request.
