# Supplementary material for: Genetically encoded formaldehyde sensors inspired by a protein intra-helical crosslinking reaction
Source: Nat Commun. 2021 Jan 25;12:581. doi: 10.1038/s41467-020-20754-4 (PMC7835342; doi:10.1038/s41467-020-20754-4)
Supplement: Supplementary file 1 — Supplementary Information [file 41467_2020_20754_MOESM1_ESM.pdf]

Supplementary Information

Genetically encoded formaldehyde sensors inspired by a protein intra-helical crosslinking reaction

Rongfeng Zhu[1,5,8], Gong Zhang[1,6,8], Miao Jing[2,3,7], Yu Han[1], Jiaofeng Li[1], Jingyi Zhao[1], Yulong Li[2,3] and Peng R. Chen[1,2,4]*

Affiliations:

[1]Synthetic and Functional Biomolecules Center, Beijing National Laboratory for Molecular Sciences, College of Chemistry and Molecular Engineering, Peking University, 100871 Beijing, China

[2]Peking-Tsinghua Center for Life Sciences, 100871 Beijing, China

[3]State Key Laboratory of Membrane Biology, PKU-IDG/McGovern Institute for Brain Research, School of Life Sciences, Peking University, 100871 Beijing, China.

[4]Key Laboratory of Bioorganic Chemistry and Molecular Engineering of Ministry of Education, Peking University, 100871 Beijing, China

[5]Current address: State Key Laboratory of Cellular Stress Biology, Innovation Center for Cell Signaling Network, School of Life Sciences, Xiamen University, Xiamen, Fujian 361005, China

[6]Current address: Chongqing Key Laboratory of Natural Product Synthesis and Drug Research, School of Pharmaceutical Sciences, Chongqing University, Chongqing, 401331, China.

[7]Current address: Chinese Institute for Brain Research, 102206 Beijing, China

[8]These authors contributed equally to this work.

*Correspondence to: Peng R. Chen, E-mail: pengchen@pku.edu.cn.

➢ **Supplementary Notes**

Supplementary Note 1 Regulation of FA detoxification in *B. subtilis*

Supplementary Note 2 Optimization of the DNA sequence for crystallization of HxlR-WT-FA-DNA

Supplementary Note 3 LC-MS analysis of methylene bridge formation via synthetic peptides

Supplementary Note 4 Descriptions of difference between the structures of HxlR-WT and HxlR-WT-FA-DNA

Supplementary Note 5 The intermediate conformation of HxlR-K13A

Supplementary Note 6 Expanded palette of FAsor for FA imaging in different subcellular compartments

➢ **Supplementary Results**

Supplementary Figure 1 The transcription factor HxlR directly senses FA via an intra-helical methylene crosslink.

Supplementary Figure 2 Cys11 and Lys13 are essential for the response of HxlR to FA.

Supplementary Figure 3 Structural investigation of FA-induced conformational change on HxlR.

Supplementary Figure 4 Development and characterization of FAsors.

Supplementary Figure 5 Subcellular visualization of FA by FAsors in living cells.

Supplementary Figure 6 Visualization of endogenous FA dynamics in living cells by FAsor

Supplementary Figure 7 Demonstration of the applicability of two-photon microscopy and AAV infection of FAsors.

Supplementary Table 1 Data collection and refinement statistics of the crystal structures.

Supplementary Table 2 Oligonucleotides used in this study.

Supplementary Table 3 Nucleotides sequence of the synthesized codon-optimized HxlR gene.

➢ **Supplementary References**

Supplementary Notes

Supplementary Note 1: Regulation of FA detoxification in *B. subtilis*

In *B. subtilis*, HxlR protein regulated the RuMP pathway to serves it as detoxification system of FA. HxlR induces transcription of downstream *hxlAB* operon, which consists of *hxlA* and *hxlB* genes and encodes two key enzymes in the RuMP pathway under stimulation of FA[1] (**Supplementary Fig. 1a**). These two enzymes, *hxlA*-encoded 3-hexulose-6-phosphate synthase (HPS) and *hxlB*-encoded 6-phospho-3-hexuloisomerase (PHI), together catalyze detoxification of FA via assimilating it into fructose 6-phosphate (**Supplementary Fig. 1a**) [1]. RuMP pathway is the major route for FA detoxification in *B. subtilis*[2]. In the absence of *hxlR* gene, expression of HPS and PHI were disrupted and cells are more sensitive to FA, indicating HxlR act as an activator but not a derepressor[1].

Besides HxlR-regulated RuMP pathway, *B. subtilis* also utilize thiol-dependent alcohol dehydrogenase AdhA for detoxification of FA, which is controlled by transcription factor AdhR[2]. Deletion of *adhR* led to disrupted transcription of *adhR*, indicating that AdhR is also an activator protein[2]. We chose HxlR for further investigation as *ΔhxlR* strain appeared to be more sensitive to FA than *ΔadhA* strain[2]. Previous work has identified HxlR-binding promoter region, while the molecular mechanism underlying HxlR's FA sensing remains unknown[3].

Supplementary Note 2: Optimization of the DNA sequence for crystallization of HxlR-WT-FA-DNA

DNA of various sequences were attempted in crystallization, which have different length from 19 bp to 25 bp in the BRH-1 sequence from the native *hxlAB* promoter sequence[3]. In order to enhance the diffraction of the crystals, palindromic sequences were generated for screening. The sequence of DNA generating crystals of HxlR-WT-FA-DNA complex that has the best diffraction was found to be palindromic blunt-ended 20 bp 5'- CAG TAT CCT CGA GGA TAC TG -3'.

Supplementary Note 3: LC-MS analysis of methylene bridge formation via synthetic peptides

Our LC-MS analysis detected a newly formed +12 Da mass peak on the synthetic peptide (Ac-FNCEKEL-NH_2) after FA incubation, confirming that FA is able to induce intramolecular crosslinking with a methylene bridge formation in this peptide (**Fig. 1d**). A +30 Da mass peak also appeared after FA incubation, which was likely corresponding to the hydroxymethylated cysteine as a reaction intermediate (**Supplementary Fig. 1c**). Notably, when Cys11 was replaced by Ala (C11A), both the +12 Da peak and the +30 Da peak disappeared (**Fig. 1d**), indicating that Cys11 is required for this intramolecular crosslinking. Meanwhile, when Lys13 was replaced by Ala (K13A), the +12 Da peak disappeared while the +30 Da mass peak corresponding to the hydroxymethylated Cys11 still remained (**Fig. 1d**). Based on these results, we propose the following interaction mechanism between FA and HxlR: FA first reacts with the thiol group on

Cys11 to form a hydroxymethylated cysteine, which subsequently reacts with the nearby amine group on Lys13 to generate an intra-helical methylene bridge (**Fig. 1c**). This mechanism is in consistent with the previous research on reactions of FA with cysteine and glutathione, in which FA also showed higher reactivity towards thiol group[4-6].

Supplementary Note 4: Descriptions of difference between the structures of HxlR-WT and HxlR-WT-FA-DNA

The major difference between HxlR-WT and HxlR-WT-FA-DNA is the HxlR-DNA interactions in HxlR-WT-FA-DNA which are absence in HxlR-WT. A total of 21 residues on HxlR that gives approximately 33 hydrogen bond or water-mediated interactions with the 20 bp palindromic operator DNA (**Supplementary Fig. 3a**). The recognition helices α4s took the main role of interactions with bases in the DNA. Direct hydrogen bond was formed between the amine group of Lys53's sidechain and O6 atom of Gua14' in the DNA, and water-mediated hydrogen bonds were formed between Lys53 and Cyt7, Gua13' (**Supplementary Fig. 3b**). Gln52' is also able to form hydrogen bond with Gua3 (**Supplementary Fig. 3c**). In addition to the interactions on base pair, HxlR also interacts with DNA's phosphate backbone. Sidechains of residues Arg39, Asn41, Arg60, Arg70 and Tyr82 and the mainchain amide group of Val80 form hydrogen bonds with the phosphate groups near the 5' end of the DNA (**Supplementary Fig. 3d**). Arg60 also have water-mediated hydrogen bond interaction with the OP1 atom in Thy4 (**Supplementary Fig. 3d**).

For overall conformation of HxlR protein, a large conformational change was observed between HxlR-WT and HxlR-WT-FA-DNA (r.m.s.d. = 2.784 Å for 188 Cα atoms in dimer, **Supplementary Fig. 3e**).

Supplementary Note 5: The intermediate conformation of HxlR-K13A

Since HxlR-K13A showed enhanced DNA binding and transcriptional activation than HxlR-WT (**Fig. 1e, Supplementary Fig. 2d,e**), we solved the structure of this mutant that was refined to 1.7-Å resolution (**Supplementary Fig. 3g and Supplementary Table 1**). Despite the similar overall architecture between HxlR-K13A and HxlR-WT, the distance between the two DNA-binding helices α4 and α4′ are shortened to 39.4 Å in HxlR-K13A, which represents a more optimal conformation for DNA-binding (**Fig. 2e**). We further superposed one subunit of HxlR dimer in HxlR-WT, HxlR-K13A and HxlR-WT-FA-DNA together, which showed that helix α4 was rotated 7.7 degree and the tips of the β-wings were translocated 8.6 Å in HxlR-K13A when compared with HxlR-WT, which are less significant changes than that of HxlR-WT-FA-DNA (**Supplementary Fig. 3h**). HxlR-K13A's more optimized conformation for DNA-binding explained the reason why HxlR-K13A has a higher basal level on DNA-binding and transcriptional activation than that of HxlR-WT.

Supplementary Note 6: Expanded palette of FAsor for FA imaging in different subcellular compartments

We also employed the recently developed circularly permuted red fluorescent proteins (cpRFPs) and constructed FAsorRed, which would have lower phototoxicity and greater tissue penetration[7]. By replacing cpYFP with circularly permuted mApple in FAsor, the resulting sensor FAsorRed showed red-shifted spectrum and higher dynamic range to FA treatment (2.7-fold vs. 2.1-fold increase for FAsorRed and FAsor in the presence of 1 mM FA; **Supplementary Fig. 4n,o**). Time-course multicolor imaging of FA concentration change in different subcellular regions with FAsor and FAsorRed indicated that FAsorRed also showed higher dynamic range towards FAsor in the cellular environment (**Supplementary Fig. 5f,g**).

Supplementary Results

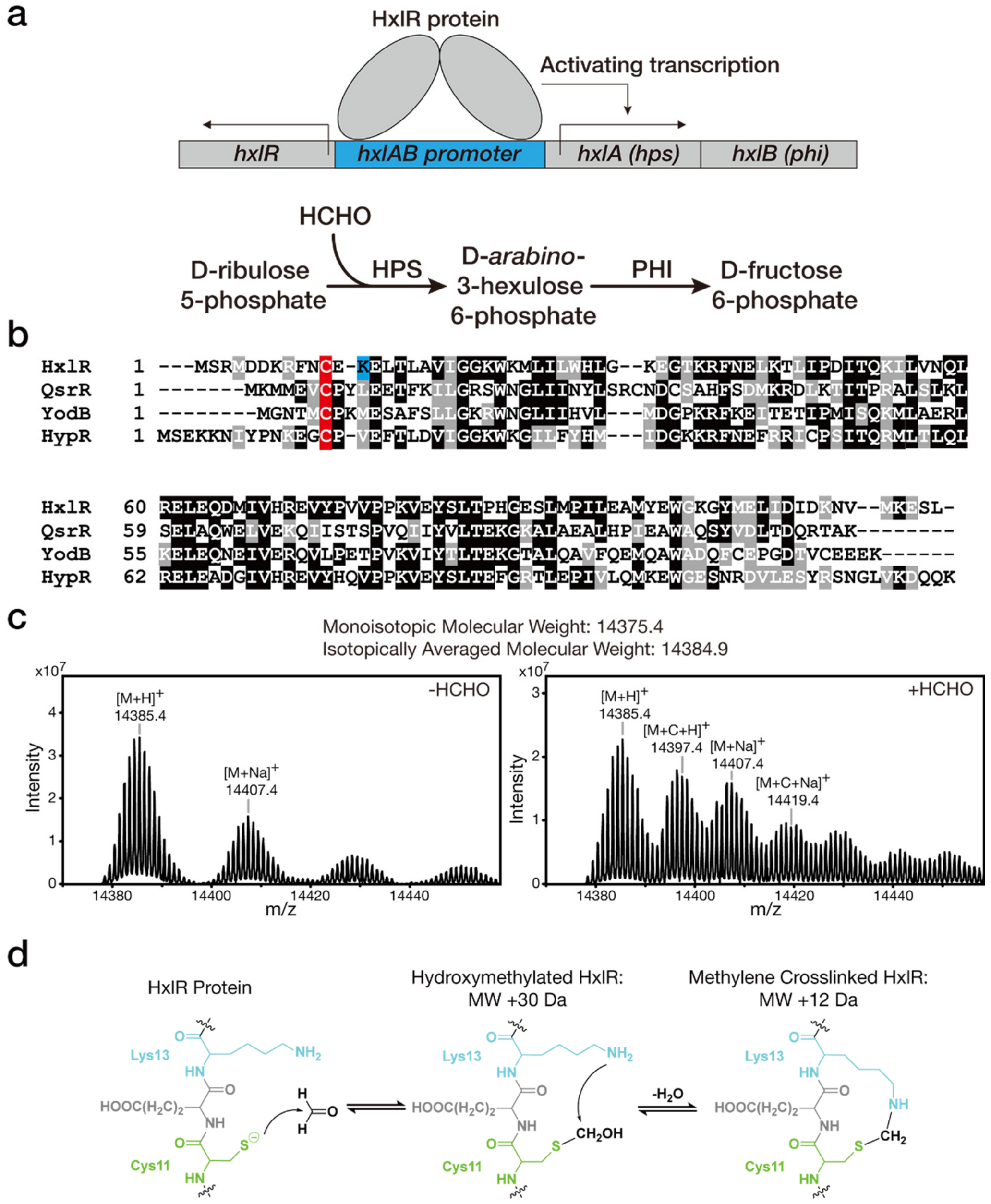

Supplementary Figure 1 | HxlR directly senses FA via the intra-helical Cys-Lys crosslinking reaction. (**a**) Operon structure of *hxlAB* and mechanism of FA detoxification by HxlA (HPS) and HxlB (PHI). (**b**) Sequence alignments of DUF24 family proteins. The sequence alignment of HxlR with selected DUF24 family members QsrR, YodB and HypR indicates that Cys11 is a conserved

residue (shaded in red). Lys13 of HxlR, which is also the key residue in FA-sensing, is shaded in cyan. Sequence alignment was shaded with BOXSHADE. (**c**) FA activates HxlR protein via formation of an intra-subunit methylene bridge. Deconvoluted FT-MS spectra of HxlR proteins indicate that +12 Da mass peaks appear after FA treatment. The +12 Da molecular weight suggests the addition of a methylene bridge between C11 and K13 as observed in the crystal structures in Fig. 1a. (**d**) Proposed reaction model of HxlR with FA. FA first reacts with the thiol group in Cys11 to form hydroxymethylated cysteine as the intermediate (MW +30 Da). The primary amine group in Lys13 then reacts with this hydroxymethyl group to form the crosslink (MW +12 Da).

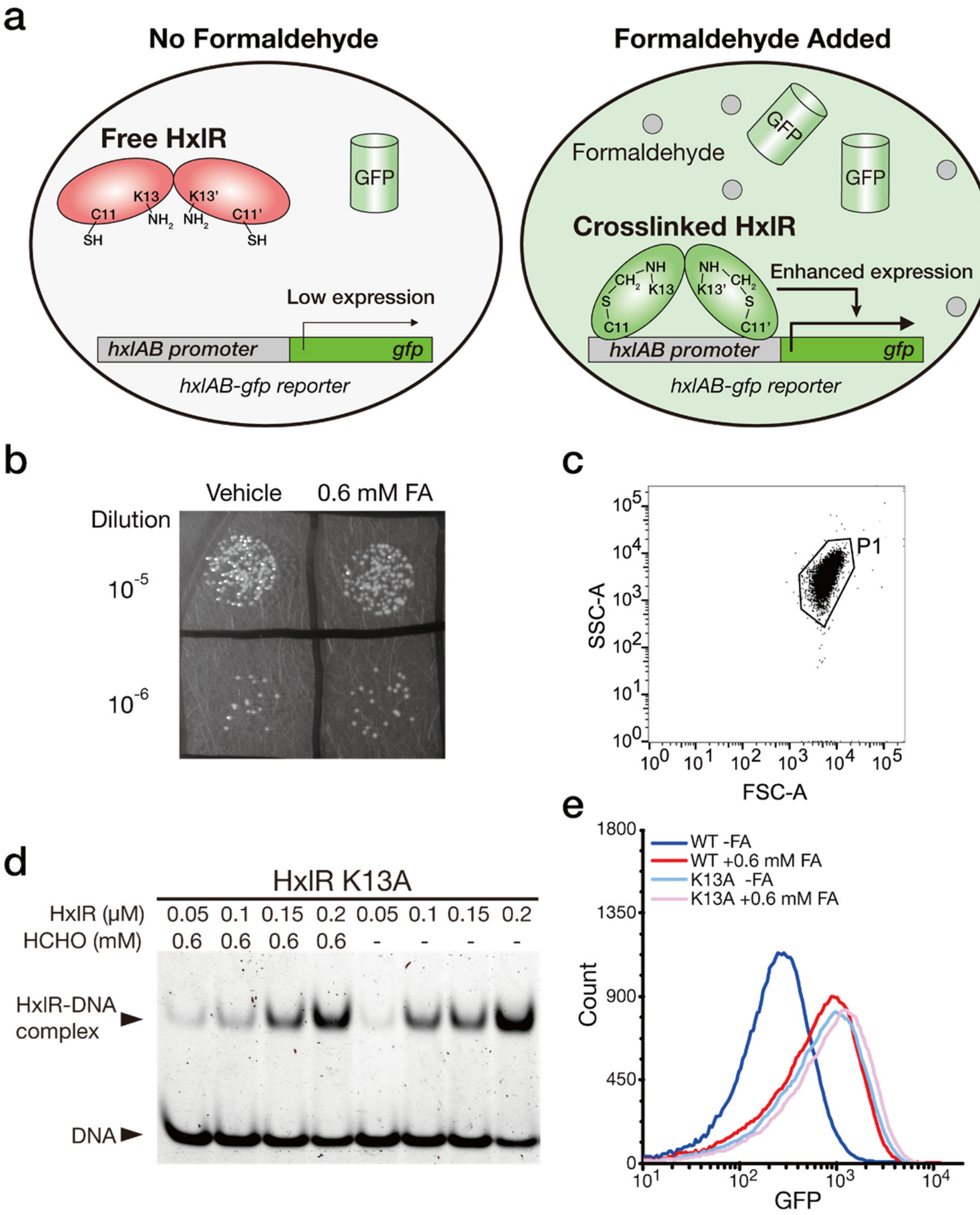

Supplementary Figure 2 | Cys11 and Lys13 are essential for the response of HxlR to FA. (a) Cartoon description for validating HxlR-controlled transcriptional activation via the *hxlAB-gfp* reporter inside cells. Transcription of *gfp* gene under the control of *hxlAB* promoter will be activated by HxlR protein, resulting in increased fluorescence intensity in living *E. coli* cells that can be analyzed by flow cytometry. Upon FA treatment, intramolecular crosslinking will induce HxlR to have enhanced transcriptional activation on the *hxlAB-gfp* reporter, which results in

increased fluorescence intensity. (**b**) 0.6 mM FA affected little on the viability of *E. coli*. *E. coli* with or without treatment of 0.6 mM FA were serially diluted and plated. Little difference of the colony counts indicated that 0.6 mM FA had negligible effect on the viability of bacteria. (**c**) Gating strategies for the FACS measurements of HxlR-regulated GFP expression. Plot gating (P1) for live cells. (**d**) The K13A mutation impairs FA-enhanced DNA-binding of HxlR protein. Determination of HxlR's DNA binding ability via EMSA showed that 0.6 mM FA was unable to increase DNA-binding ability of HxlR-K13A. Similar results were obtained from 3 independent experiments. (**e**) Flow cytometry analysis of the *hxlAB-gfp* reporter-harbored *E. coli* BW25113 cells expressing HxlR-WT or HxlR-K13A protein with and without FA treatment. Cells were treated with 0.6 mM FA for 40 min before being analyzed by flow cytometry. Addition of FA increased the GFP expression level in the HxlR-WT-expressing strain but not the HxlR-K13A expressing strain.

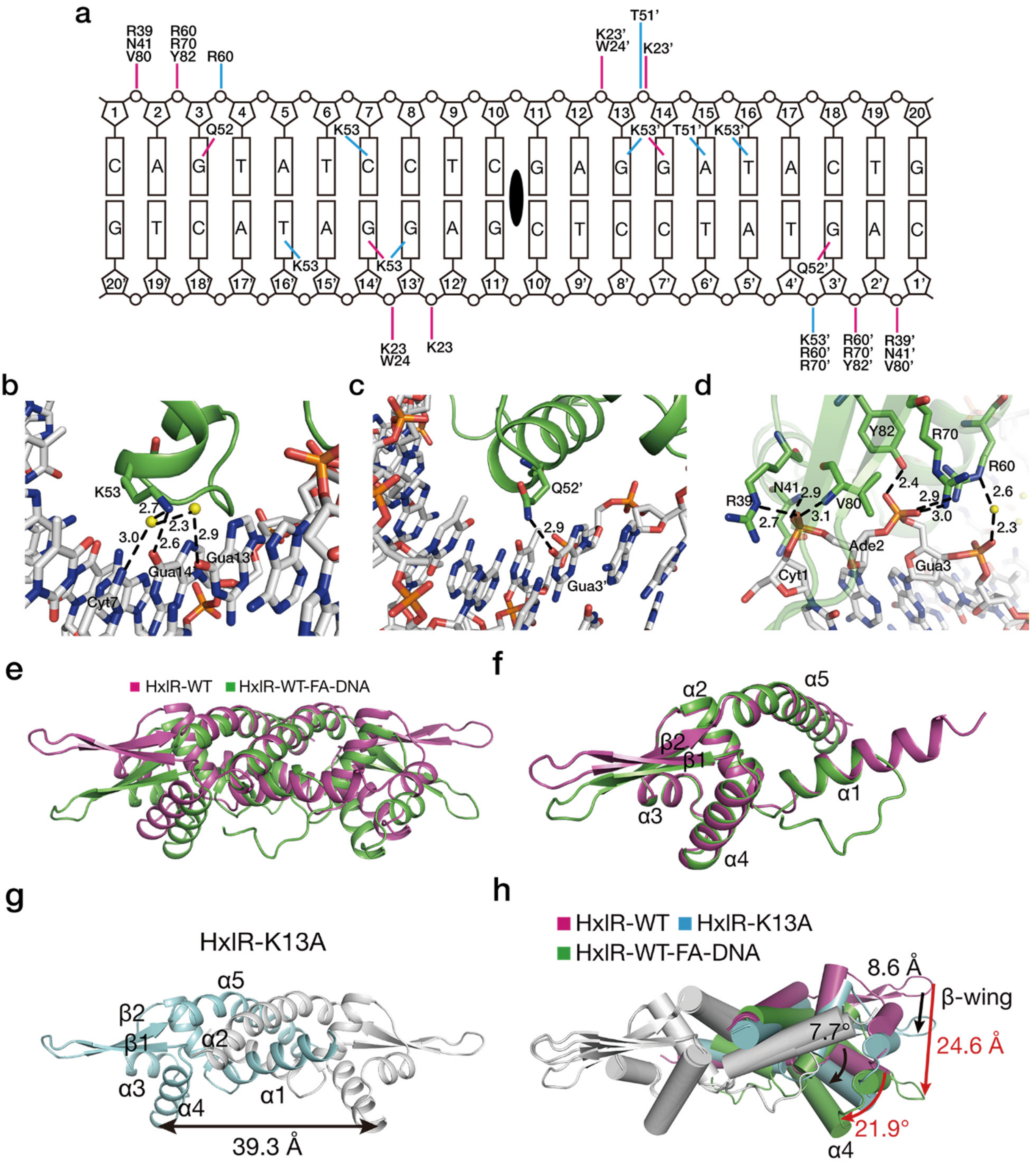

Supplementary Figure 3 | Structural investigation of FA-induced conformational change on HxlR. (**a**) Schematic representation of the interactions of HxlR with its operator DNA. Cyan lines indicate water-mediated interactions between HxlR protein and DNA, and magenta lines indicate direct electrostatic interactions between HxlR protein and DNA. (**b**) The close view of the

interactions between the Lys53 and the operator DNA base pairs. (**c**) The close view of the interactions between the Gln52' and the operator DNA base pairs. (**d**) The close view of the interactions between HxlR and phosphate backbones of DNA near the 5' end. In (**b-d**) water molecules are shown as yellow spheres, and distances of putative hydrogen bonds are labeled in angstrom units. (**e**, **f**) HxlR-WT and HxlR-WT-FA-DNA take large difference when superposed in dimer (**e**)(r.m.s.d. = 2.784 Å for 188 Cα atoms in dimer), but fit close to each other when superposed in monomer (**f**)(r.m.s.d. = 0.595 Å for 82 Cα atoms). (**g**) Crystal structure of HxlR-K13A protein. HxlR-K13A have a similar overall architecture with HxlR-WT, which consists of 5 α-helices and 2 antiparallel β-strands. The distance between the DNA-binding helices α4 and α4′ is shortened to 39.4 Å. (**h**) HxlR-K13A takes an intermediate conformation between HxlR-WT-FA-DNA and HxlR-WT. Superposition of HxlR-WT (magenta), HxlR-K13A (cyan) and HxlR-WT-FA-DNA (green) by Cα atoms in one subunit illustrated that FA activation or K13A mutation induce HxlR to take a more optimized conformation for DNA-binding. Red arrows indicate rotation and translocation of wHTH domain in HxlR-WT upon FA activation. Black arrows indicate rotation and translocation of wHTH domain induced by K13A mutation, which results in a conformation between that of HxlR-WT and HxlR-WT-FA-DNA.

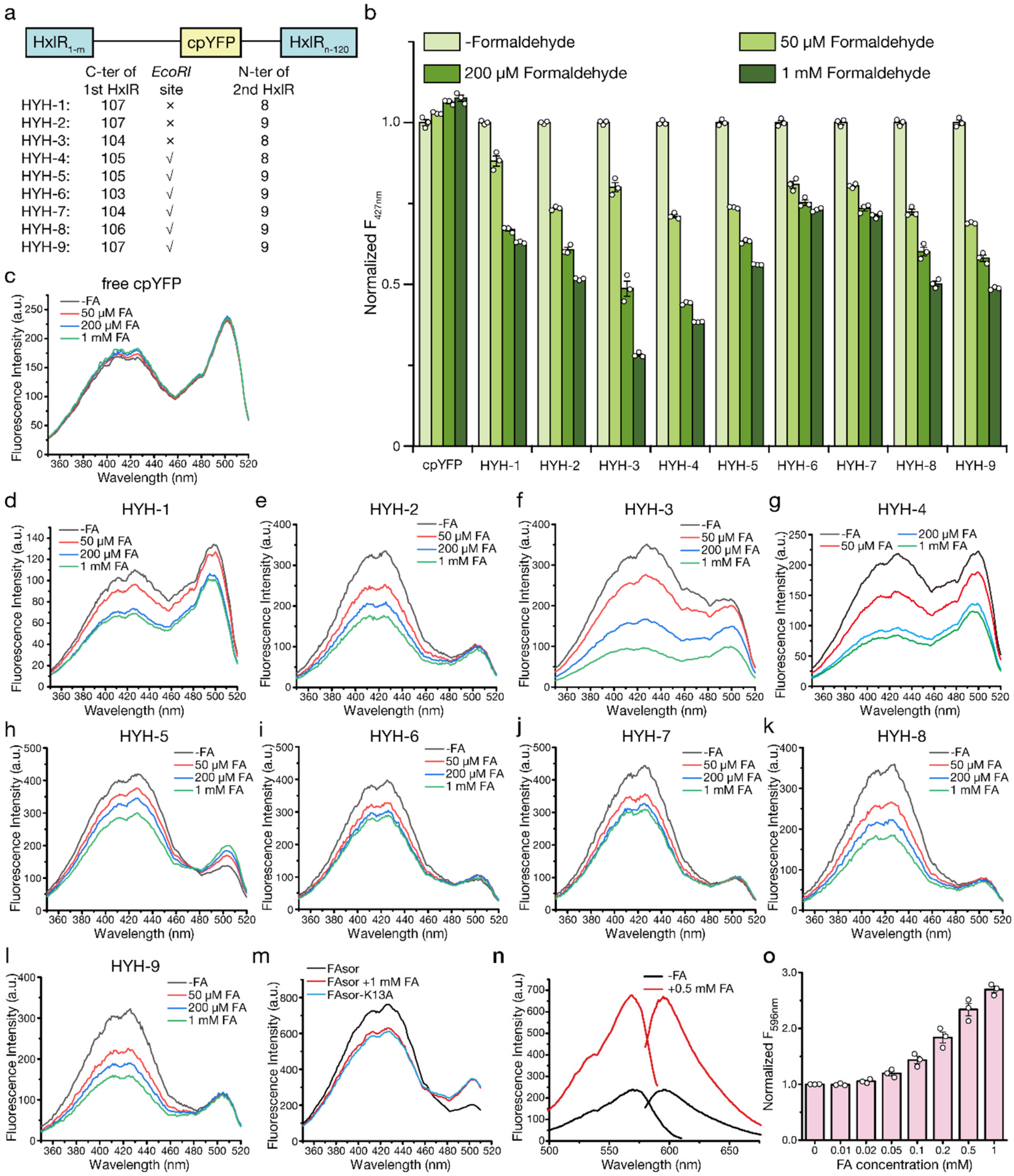

Supplementary Figure 4 | Development and characterization of FAsors. (**a,b**) Summary of the fluorescence performance of tested HxlR-cpYFP-HxlR constructions. Different constructs (**a**) were tested with their performance shown in (**b**). All of the 9 HxlR-cpYFP-HxlR constructions showed dose-dependent fluorescence response upon addition of FA whereas cpYFP alone showed little change, indicating that HxlR-cpYFP-HxlR constructions reported the conformational change

of HxlR upon reaction with FA. Data are shown in mean ± SEM for 3 measurements. (**c-l**) Excitation spectra of different HxlR-cpYFP-HxlR constructions after the treatment of different concentrations of FA. Only HYH-5 (**h**) has an increased fluorescence response to FA when excited above 467 nm, while all other constructions showed decreased fluorescence response. Emission filter was fixed at 530 nm. (**m**) Comparison of the excitation spectra of FAsor-K13A with FAsor-WT with/without 1 mM FA treatment. Emission filter was fixed at 516 nm. (**n**) Excitation and emission spectra of FAsorRed in the absence (black) and presence (red) of 0.5 mM FA. The excitation peak and emission peak of FAsorRed are 569 nm and 594 nm, respectively. FAsorRed exhibited a turn-on response to FA, with its fluorescence increased upon FA treatment. (**o**) The fluorescence change of FAsorRed in response to a serial concentration of FA ranging from 0 to 1 mM in 30 min. The ratio is calculated by dividing the fluorescence intensity of FAsorRed with the untreated sample. Data are shown in mean ± SEM for 3 measurements.

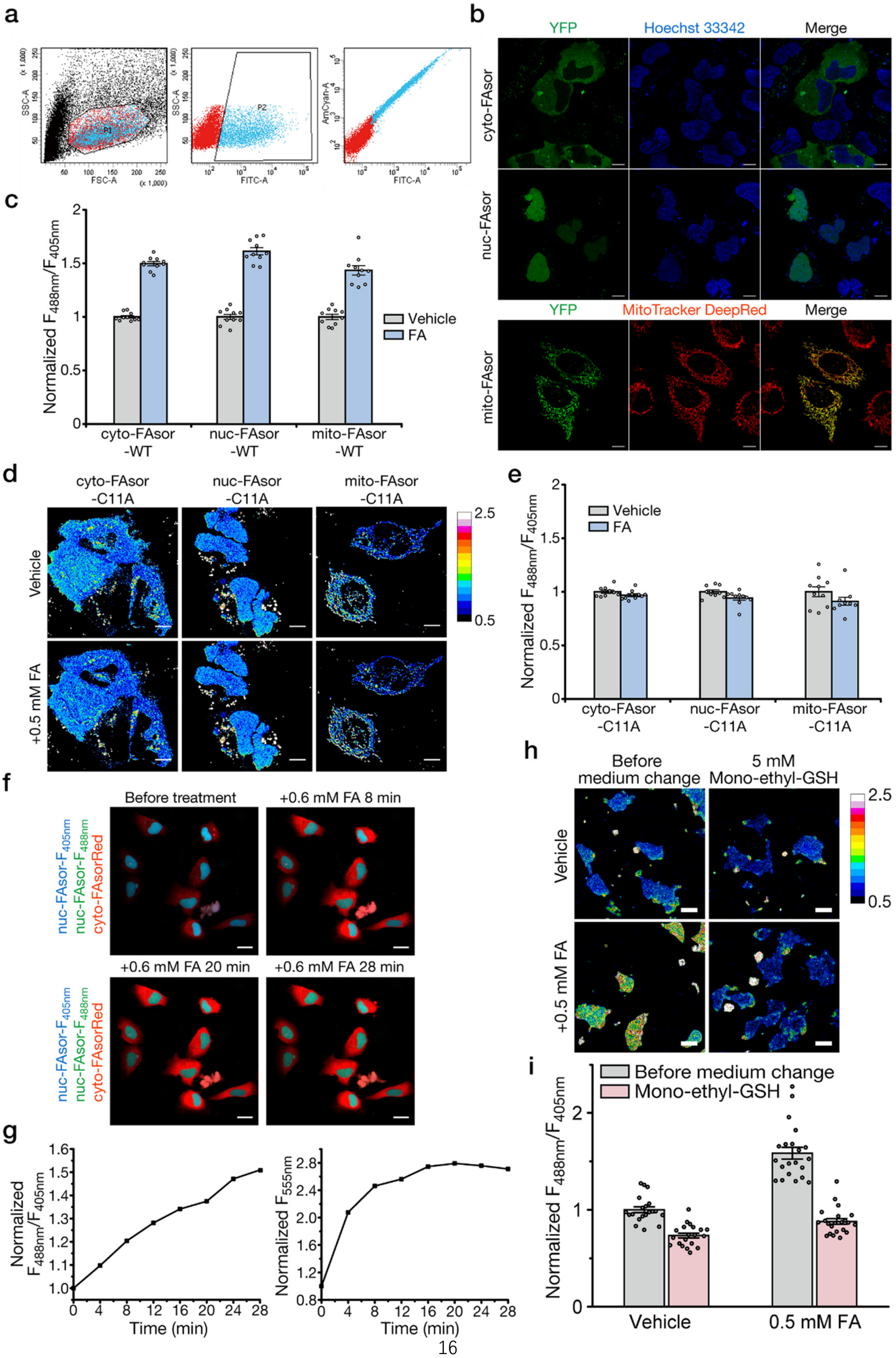
a
SSC-A
FSC-A
P1
P2
FITC-A
AmCyan-A
b
YFP
Hoechst 33342
Merge
cyto-FAsor
nuc-FAsor
YFP
MitoTracker DeepRed
Merge
mito-FAsor
c
Normalized F488nm/F405nm
Vehicle
FA
cyto-FAsor -WT
nuc-FAsor -WT
mito-FAsor -WT
d
cyto-FAsor -C11A
nuc-FAsor -C11A
mito-FAsor -C11A
Vehicle
+0.5 mM FA
2.5
0.5
e
Normalized F488nm/F405nm
Vehicle
FA
cyto-FAsor -C11A
nuc-FAsor -C11A
mito-FAsor -C11A
f
Before treatment
+0.6 mM FA 8 min
+0.6 mM FA 20 min
+0.6 mM FA 28 min
nuc-FAsor-F405nm
nuc-FAsor-F488nm
cyto-FAsorRed
g
Normalized F488nm/F405nm
Time (min)
Normalized F555nm
Time (min)
h
Before medium change
5 mM Mono-ethyl-GSH
Vehicle
+0.5 mM FA
2.5
0.5
i
Before medium change
Mono-ethyl-GSH
Normalized F488nm/F405nm
Vehicle
0.5 mM FA

Supplementary Figure 5 | Subcellular visualization of FA by FAsors in living cells. (**a**) Gating strategies for the FACS measurements of FAsor fluorescence. First plot gating (P1) for live cells, then second plot for (P2) FAsor expressing cells. (**b**) Localization of subcellular-targeting FAsor constructs. The cytosolic FAsor and nuclear FAsor were expressed in HeLa cells, with the staining of Hoechst 33342 showing cell nucleus. The mitochondrial FAsor was expressed in HeLa cells, with the staining of MitoTracker DeepRed, showing good colocalization. Scale bars, 10 μm. (**c**) Statistical analysis of subcellular visualization of FAsor-WT. Each group contains n = 10 cells. (**d**) Subcellular visualization of the FAsor-C11A mutant in living cells. The subcellular-targeted FAsor-C11A constructs were expressed in cells, followed by the treatment of 0.5 mM FA. The calculated F_{488nm}/F_{405nm} ratio showed little change, ruling out the possibility of environmental pH changes in measurement of FAsor response to FA. Scale bars, 10 μm. (**e**) Statistical analysis of subcellular visualization of FAsor-C11A. Each group contains n = 10 cells. (**f**) Time-course multicolor imaging of FA concentration change in different subcellular regions with FAsor and FAsorRed. Nucleus located FAsor and cytoplasm located FAsorRed are co-transfected into HeLa cells. Cells expressing FAsor and FAsorRed were treated with 0.6 mM FA for 30 min. Fluorescence of FAsor were excited at 405 nm (blue) and 488 nm (green), and fluorescence of FAsorRed was excited at 555 nm (red). Scale bars, 20 μm. (**g**) Fluorescence change of subcellular-targeted FAsor and FAsorRed proteins in (**f**). (**h**) Fluorescence imaging of FAsor in response to GSH. FAsor without subcellular targeting sequence were expressed in cells, followed by the treatment of no FA or 0.5 mM FA for 30 min. The cells were next changed to medium with 5 mM mono-ethyl-GSH added and incubated for another 30 min. Scale bars, 20 μm. (**i**) Statistical analysis of visualization of FAsor in response to GSH. For groups from left to right, n=20, 20, 21 and 22 cells respectively. In (**d, h**), images were pseudocolored with normalized F_{488nm}/F_{405nm} ratio. Data in (**c, e, i**) are shown in mean ± SEM.

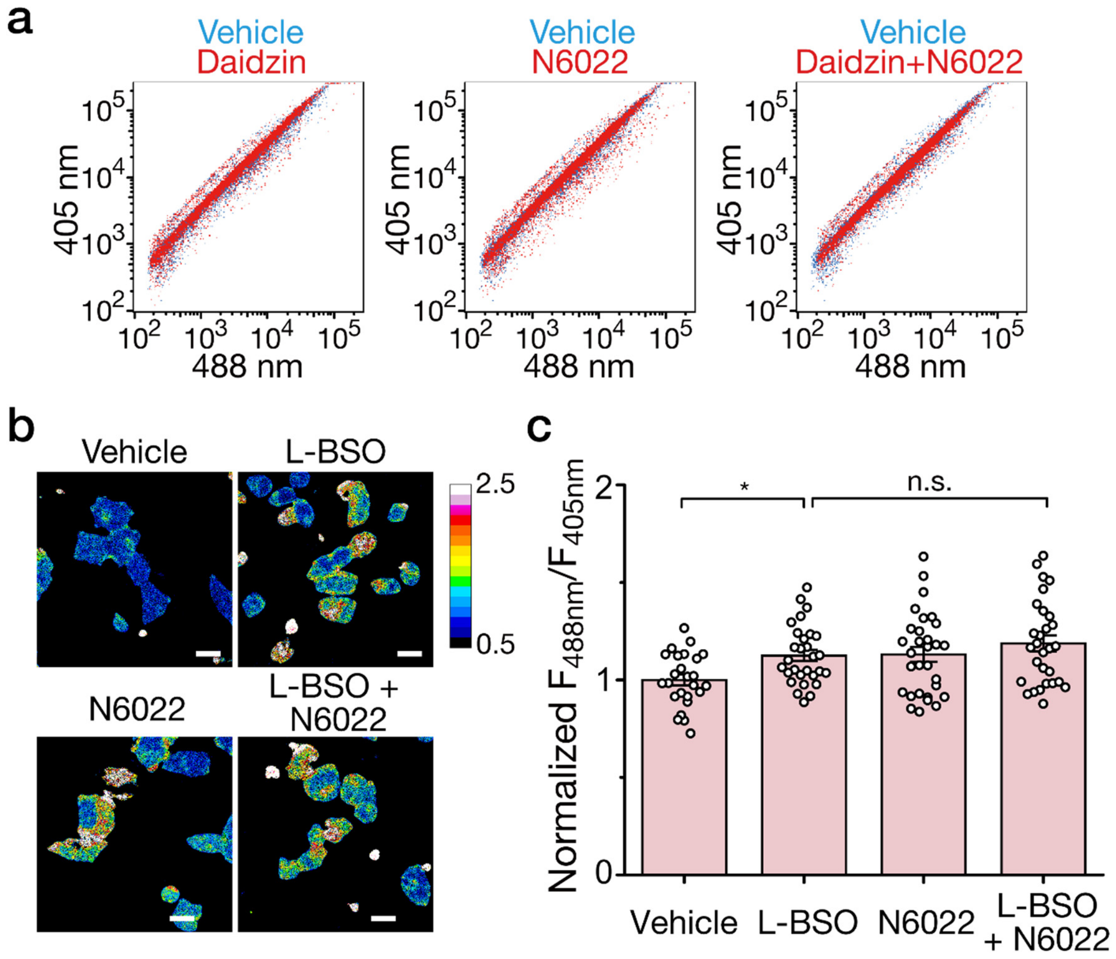

Supplementary Figure 6 | Visualization of endogenous FA dynamics in living cells by FAsor. (**a**) FACS measurements of ratio of fluorescence excited at 488 nm and 405 nm of FAsor in response to FA in different states of FA metabolism. HEK293T cells are incubated with different inhibitors (10 μM each) at 37 °C for 3 hours. (**b**) Fluorescence imaging of FAsor in response to L-BSO. FAsor without subcellular targeting sequence were expressed in cells, followed by the treatment of no L-BSO or 100 μM L-BSO for 18 hours. The medium was next added with or without 10 μM N6022 and the cells were incubated for another 3 hours. Scale bars, 20 μm. Images were pseudocolored with normalized F_{488nm}/F_{405nm} ratio. (**c**) Statistical analysis of subcellular visualization of FAsor in response to L-BSO. For groups from left to right, n=25, 29, 30 and 29 cells respectively. Data are shown in mean ± SEM. n.s., no significance (P=0.21). *, P<0.01 (P=0.0026).

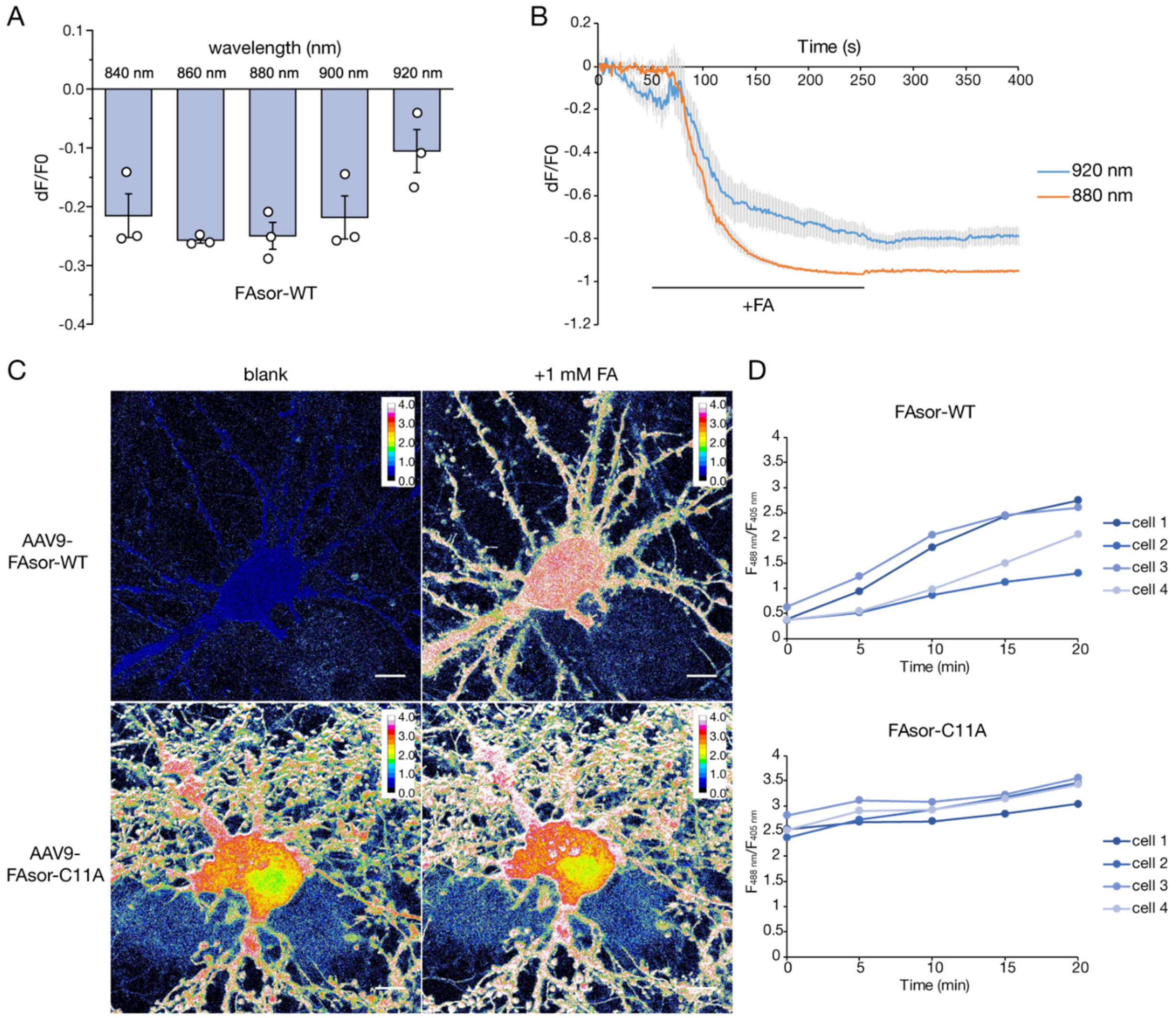

Supplementary Figure 7 | Demonstration of the applicability of two-photon microscopy and AAV infection of FAsors. (**a**) Response of FAsor to FA under two-photon excitation microscopy. The fluorescence intensity changes of FAsor protein (20 μM) treated with 2 mM FA *in vitro*, under two-photon excitation of different wavelengths. Data are presented as mean ± SEM for 3 measurements. (**b**) The fluorescence intensity change of FAsor-WT was monitored continuously with 1 mM FA perfusion treatment in HeLa cells, with two-photon excitation wavelength set as 920 nm or 880 nm. Time of FA treatment was indicated by the line. Curves represent quantification data of n = 6 cells. Data are presented as mean ± SD. The FAsor-WT showed a turn-off intensiometric property under both 920 nm and 880 nm two-photon excitation. (**c**) Response of

FAsor-expressing cultured neurons to the treatment of FA. Adeno-associated virus AAV9 carrying FAsor was used to infect cultured neurons. The AAV9-FAsor could be successfully expressed in cultured neurons after 3 days infection, and the $F_{488\ nm}/F_{405\ nm}$ ratio indicates significant change of the FA level upon treatment with 1 mM FA. Scale bars, 10 μm. Similar result were obtained from 4 independent experiments. (**d**) Fluorescence change of cultured neurons at different time points upon FA treatment.

Supplementary Table 1: Data collection and refinement statistics of the crystal structures of HxlR

	**HxlR-FA-DNA**	**HxlR-WT**	**HxlR-K13A**
**Data collection**			
Space group	$P2_1$	$I222$	$P2_12_12$
Cell dimensions			
a, *b*, *c* (Å)	56.1, 109.3, 160.4	53.2, 60.8, 70.7	60.8, 70.1, 28.3
α, β, γ (°)	90, 99.8, 90	90, 90, 90	90, 90, 90
Resolution (Å)	50.0-2.90 (2.95-2.90)	50.0-2.60 (2.64-2.60)	50.0-1.66 (1.69-1.66)
R_{merge}	0.095(0.966)	0.065(0.816)	0.063(0.882)
$I / \sigma I$	17.6(2.0)	28.7(3.3)	30.4(2.1)
Completeness (%)	99.8(100.0)	99.7(100.0)	98.8(98.9)
Redundancy	3.8(3.8)	6.8(7.1)	6.2(5.7)
**Refinement**			
Resolution (Å)	33.41-2.90	46.08-2.61	30.36-1.66
No. reflections	42357	3662	14728
R_{work} / R_{free}	0.179/0.223	0.241/0.271	0.204/0.220
No. atoms			
Protein	5436	908	893
DNA	2442	0	0
Ligand/ion	95	0	14
Water	178	0	64
B-factors			
Protein	67.86	84.45	41.93
DNA	63.55	-	-
Ligand/ion	84.08	-	56.15
Water	61.04	-	51.59
R.m.s. deviations			
Bond lengths (Å)	0.005	0.009	0.014
Bond angles (°)	0.699	1.194	1.332

Numbers in parentheses are values for highest-resolution shell.

Ramachandran statistics, wavelength of data collection, temperature and beamline are reported in materials and methods section.

Supplementary Table 2: Oligonucleotides used in this study

	Oligonucleotide Sequence (5'-3')
For plasmid construction	
pET28a-HxlR-F	GGAATTCCATATGAGCCGGATGGACGACAAAAGG
pET28a-HxlR-R	CCGCTCGAGTCACAACGATTCTTTCATGAC
pBAD-HxlR-F	CATGCCATGGGCCGGATGGACGACAAAAGG
pBAD-HxlR-R	CCCAAGCTTTCACAACGATTCTTTCATGAC
pET28a-HYH-cpYFP-105eF	GAGTGGGGGAAAGGCTATGAATTTTACAACAGCGACAACGTCTATATC
pET28a-HYH-cpYFP-9R	CGTTAATTCCTTCTCACAATTAAAGTTGTACTCCAGCTTGTGCCCCAG
pET28a-HYH-2ndHxlR-9F	CTGGGGCACAAGCTGGAGTACAACTTTAATTGTGAGAAGGAATTAACG
pET28a-HYH-2ndHxlR-R	GTGCTCGAGTCACAACGATTCTTTCATGACATTTTTGTCG
pET28a-HYH-vector-F	CGACAAAAATGTCATGAAAGAATCGTTGTGACTCGAGCAC
pET28a-HYH-vector-105eR	GATATAGACGTTGTCGCTGTTGTAAAATTCATAGCCTTTCCCCCACTC
pET28a-HYH-2ndHxlR-8F	CTGGGGCACAAGCTGGAGTACAACAGGTTTAATTGTGAGAAGG
pET28a-HYH-cpYFP-8R	CCTTCTCACAATTAAACCTGTTGTACTCCAGCTTGTGCCCCAG
pET28a-HYH-cpYFP-104F	CATGTATGAGTGGGGGAAAGGCTACAACAGCGACAACGTCTATATC
pET28a-HYH-cpYFP-107F	GAGTGGGGGAAAGGCTATATGGAATACAACAGCGACAACGTCTATATC
pET28a-HYH-vector-104R	GATATAGACGTTGTCGCTGTTGTAGCCTTTCCCCCACTCATACATG
pET28a-HYH-vector-107R	GATATAGACGTTGTCGCTGTTGTATTCCATATAGCCTTTCCCCCACTC
HYH-7-mut-F	GTATGAGTGGGGGAAAGGCGAATTTTACAACAGCGACAACG
HYH-7-mut-R	CGTTGTCGCTGTTGTAAAATTCGCCTTTCCCCCACTCATAC
HYH-6-mut-F	CCATGTATGAGTGGGGGAAAGAATTTTACAACAGCGACAACG
HYH-6-mut-R	CGTTGTCGCTGTTGTAAAATTCTTTCCCCCACTCATACATGG
HYH-8-mut-F	GTATGAGTGGGGGAAAGGCTATATGGAATTTTACAACAGCGACAACG
HYH-8-mut-R	CGTTGTCGCTGTTGTAAAATTCCATATAGCCTTTCCCCCACT

	CATAC
HYH-9-mut-F	GTATGAGTGGGGGAAAGGCTATATGGAAGAATTTTACAACAGCGACAACG
HYH-9-mut-R	CGTTGTCGCTGTTGTAAAATTCTTCCATATAGCCTTTCCCCCACTCATAC
HYH-2-mut-F	GAGTGGGGGAAAGGCTATATGGAATACAACAGCGACAACGTCTATATC
HYH-2-mut-R	GATATAGACGTTGTCGCTGTTGTATTCCATATAGCCTTTCCCCCACTC
pcDNA-FAsor-1stHxlR-F	CAAGCTGGCTAGTTAAGCTTGCCACCATGGGCAGGATGGAC
pcDNA-FAsor-1stHxlR-R	GATATAGACGTTGTCGCTGTTGTAGAACTCGTAGCCCTTGCCCCACTCGTAC
pcDNA-FAsor-cpYFP-F	GTACGAGTGGGGCAAGGGCTACGAGTTCTACAACAGCGACAACGTCTATATC
pcDNA-FAsor-cpYFP-R	CAGCTCCTTCTCACAGTTGAAGTTGTACTCCAGCTTGTGCCCCAG
pcDNA-FAsor-2ndHxlR-F	CTGGGGCACAAGCTGGAGTACAACTTCAACTGTGAGAAGGAGCTG
pcDNA-FAsor-2ndHxlR-R	GCTGGATATCTGCAGAATTCCTCACAGGCTCTCCTTCATCAC
pcDNA-FAsor-vector-F	GTGATGAAGGAGAGCCTGTGAGGAATTCTGCAGATATCCAGC
pcDNA-FAsor-vector-R	GTCCATCCTGCCCATGGTGGCAAGCTTAACTAGCCAGCTTG
pcDNA-FAsorRed-cpRFP-F	GTACGAGTGGGGCAAGGGCTACGAGTTCGTTTCCGAGCGGATGTACC
pcDNA-FAsorRed-cpRFP-R	CAGCTCCTTCTCACAGTTGAAAGCCTCCCAGCCCATGGTCTTC
pcDNA-FAsorRed-2ndHxlR-F	GAAGACCATGGGCTGGGAGGCTTTCAACTGTGAGAAGGAGCTG
pcDNA-FAsorRed-1stHxlR-R	GGTACATCCGCTCGGAAACGAACTCGTAGCCCTTGCCCCACTCGTAC
FAsor-NES-F	GAGTTGGATCTGGACTCGTACAAGTGAGGAATTCTGCAGATATCCAGC
FAsor-NES-R	TTCCAGCTTGTTCTGCAGCTCACTCAGGCTCTCCTTCATCACGTTC
FAsor-NLS-F	GGGTCAAGTTGGACTGAGGAATTCTGCAGATATCCAGC
FAsor-NLS-R	TCTTGGCAGCTGGCAGGCTCTCCTTCATCACGTTC
FAsor-mito2-F	GTTGCCGCCGGAGGGGAAGCTTGGATCCGGCAGGATGGACGATAAGAGG

FAsor-mito2-R	TGAGAAAACTCTTGTAGCGAGCATGGTGGCAAGCTTAACTAGC
FAsor-mito3-F	GCGGCTCCCAGTGCCGCGCCAAGATCCATTCGTTGCCGCCGGAGGGGAAGCTTCTCGCTACAAGAGTTTTC
For EMSA	
BRH-F	CCTCACAGTATCCTCCAAGTAACTTGTTGACTTCAAAGTGCCTACTTCTC
BRH -R	GAGAAGTAGGCACTTTGAAGTCAACAAGTTACTTGGAGGATACTGTGAGG

Supplementary Table 3: Nucleotides sequence of the synthesized codon-optimized HxlR gene

Oligonucleotide Sequence (5’-3’)
ATGAGCAGGATGGACGATAAGAGGTTCAACTGTGAGAAGGAGCTGACCC<br>TGGCCGTGATCGGCGGCAAGTGGAAGATGCTGATCCTGTGGCACCTGGG<br>CAAGGAGGGCACCAAGAGGTTTAACGAGCTGAAGACCCTGATCCCCGAC<br>ATCACCCAGAAGATCCTGGTGAACCAGCTGAGGGAGCTGGAGCAGGAC<br>ATGATCGTGCACAGGGAGGTGTATCCCGTGGTGCCCCCCAAGGTGGAGT<br>ACAGCCTGACACCCCACGGCGAGAGCCTGATGCCCATCCTGGAGGCCAT<br>GTACGAGTGGGGCAAGGGCTACATGGAGCTGATCGACATCGACAAGAAC<br>GTGATGAAGGAGAGCCTGTGA

Supplementary References

1 Yasueda, H., Kawahara, Y. & Sugimoto, S.-i. *Bacillus subtilis yckG* and *yckF* Encode Two Key Enzymes of the Ribulose Monophosphate Pathway Used by Methylotrophs, and *yckH* Is Required for Their Expression. *J. Bacteriol.* **181**, 7154-7160, (1999).

2 Nguyen, T. T. *et al.* Genome-wide responses to carbonyl electrophiles in *Bacillus subtilis*: control of the thiol-dependent formaldehyde dehydrogenase AdhA and cysteine proteinase YraA by the MerR-family regulator YraB (AdhR). *Mol. Microbiol.* **71**, 876-894, (2009).

3 Yurimoto, H. *et al.* HxlR, a member of the DUF24 protein family, is a DNA-binding protein that acts as a positive regulator of the formaldehyde-inducible hxlAB operon in Bacillus subtilis. *Mol. Microbiol.* **57**, 511-519, (2005).

4 Kallen, R. G. Equilibriums for the reaction of cysteine and derivatives with formaldehyde and protons. *J. Am. Chem. Soc.* **93**, 6227-6235, (1971).

5 Naylor, S., Mason, R. P., Sanders, J. K. M., Williams, D. H. & Moneti, G. Formaldehyde adducts of glutathione. Structure elucidation by two-dimensional n.m.r. spectroscopy and fast-atom-bombardment tandem mass spectrometry. *Biochemical Journal* **249**, 573-579, (1988).

6 Hopkinson, R. J., Barlow, P. S., Schofield, C. J. & Claridge, T. D. W. Studies on the reaction of glutathione and formaldehyde using NMR. *Organic & Biomolecular Chemistry* **8**, 4915-4920, (2010).

7 Zhao, Y. X. *et al.* An Expanded Palette of Genetically Encoded Ca2+ Indicators. *Science* **333**, 1888-1891, (2011).
