# Supplementary material for: Genetically encoded formaldehyde sensors inspired by a protein intra-helical crosslinking reaction
Source: Nat Commun. 2021 Jan 25;12:581. doi: 10.1038/s41467-020-20754-4 (PMC7835342; doi:10.1038/s41467-020-20754-4)
Supplement: Supplementary file 2 — Reporting Summary [file 41467_2020_20754_MOESM2_ESM.pdf]

nature research

Corresponding author(s): Peng R. Chen, pengchen@pku.edu.cn

Last updated by author(s): Nov 25, 2020

Reporting Summary

Nature Research wishes to improve the reproducibility of the work that we publish. This form provides structure for consistency and transparency in reporting. For further information on Nature Research policies, see our Editorial Policies and the Editorial Policy Checklist.

Statistics

For all statistical analyses, confirm that the following items are present in the figure legend, table legend, main text, or Methods section.

n/a	Confirmed	
☐	☒	The exact sample size (n) for each experimental group/condition, given as a discrete number and unit of measurement
☐	☒	A statement on whether measurements were taken from distinct samples or whether the same sample was measured repeatedly
☐	☒	The statistical test(s) used AND whether they are one- or two-sided<br>*Only common tests should be described solely by name; describe more complex techniques in the Methods section.*
☒	☐	A description of all covariates tested
☒	☐	A description of any assumptions or corrections, such as tests of normality and adjustment for multiple comparisons
☐	☒	A full description of the statistical parameters including central tendency (e.g. means) or other basic estimates (e.g. regression coefficient) AND variation (e.g. standard deviation) or associated estimates of uncertainty (e.g. confidence intervals)
☐	☒	For null hypothesis testing, the test statistic (e.g. F, t, r) with confidence intervals, effect sizes, degrees of freedom and P value noted<br>*Give P values as exact values whenever suitable.*
☒	☐	For Bayesian analysis, information on the choice of priors and Markov chain Monte Carlo settings
☒	☐	For hierarchical and complex designs, identification of the appropriate level for tests and full reporting of outcomes
☒	☐	Estimates of effect sizes (e.g. Cohen's d, Pearson's r), indicating how they were calculated

Our web collection on statistics for biologists contains articles on many of the points above.

Software and code

Policy information about availability of computer code

Data collection	Blu-Ice (5.0) for X-ray diffraction data collection, Masslynx (4.1) and Xcalibur (4.0) for mass-spec data collection, ZEN (2011) for confocal imaging, BD FACSDiva (7.0) for flow cytometry data collection, Cary Eclipse Software (1.1) for in vitro fluorescence spectra collection.
Data analysis	ClustalO (1.2.4) for protein sequence alignment, HKL2000 (v706), HKL3000 (v706), CCP4 package (6.4), Phenix (1.14-3260) and COOT (0.8.9.1) for processing of diffraction data and refinement of crystal structure, PyMOL (2.3.0) for visualization of protein structure, FCS Express (v3) for analyzing flow cytometry data, ImageJ (1.52a) for processing of imaging data, Origin (2018) and MATLAB (2018b) for plotting and statistics.

For manuscripts utilizing custom algorithms or software that are central to the research but not yet described in published literature, software must be made available to editors and reviewers. We strongly encourage code deposition in a community repository (e.g. GitHub). See the Nature Research guidelines for submitting code & software for further information.

Data

Policy information about availability of data

All manuscripts must include a data availability statement. This statement should provide the following information, where applicable:

- Accession codes, unique identifiers, or web links for publicly available datasets
- A list of figures that have associated raw data
- A description of any restrictions on data availability

Atomic coordinates and structure factors for the crystal structures have been deposited with accession codes PDB ID 7BZD [http://doi.org/10.2210/pdb7bzd/pdb] for HxlR-WT, 7BZE [http://doi.org/10.2210/pdb7bze/pdb] for HxlR-K13A and 7BZG [http://doi.org/10.2210/pdb7bzg/pdb] for HxlR-WT-FA-DNA, respectively. The PDB accession code 4A5N [https://www.rcsb.org/structure/4A5N] corresponding to the HypR protein and PDB accession code 4HQE [https://www.rcsb.org/structure/4HQE] corresponding to the QsrR-DNA complex was used in this study. The UniProt accession codes P42406 [https://www.uniprot.org/uniprot/P42406] was also used in this study. Other data are available from the corresponding author upon reasonable request. Source data are provided with this paper.

Field-specific reporting

Please select the one below that is the best fit for your research. If you are not sure, read the appropriate sections before making your selection.

☒ Life sciences ☐ Behavioural & social sciences ☐ Ecological, evolutionary & environmental sciences

For a reference copy of the document with all sections, see nature.com/documents/nr-reporting-summary-flat.pdf

Life sciences study design

All studies must disclose on these points even when the disclosure is negative.

Sample size	No statistical methods were used to predetermine the sample sizes. Sample sizes are indicated for each experiment and were chosen based on similar studies.
Data exclusions	There were no data exclusions.
Replication	All experiments were confirmed with multiple biological replicates as indicated in the Figure legends, and the representative results are shown.
Randomization	Animals or cells were randomly assigned into control or experimental groups.
Blinding	No blinding was carried out in all the experiments. No human participant was included in this study and most experiments did not use animal. For imaging of tissue slices, the experimental conditions were obvious and the analyses were performed objectively.

Reporting for specific materials, systems and methods

We require information from authors about some types of materials, experimental systems and methods used in many studies. Here, indicate whether each material, system or method listed is relevant to your study. If you are not sure if a list item applies to your research, read the appropriate section before selecting a response.

Materials & experimental systems

n/a	Involved in the study	
☒	☐	Antibodies
☐	☒	Eukaryotic cell lines
☒	☐	Palaeontology and archaeology
☐	☒	Animals and other organisms
☒	☐	Human research participants
☒	☐	Clinical data
☒	☐	Dual use research of concern

Methods

n/a	Involved in the study	
☒	☐	ChIP-seq
☐	☒	Flow cytometry
☒	☐	MRI-based neuroimaging

Eukaryotic cell lines

Policy information about cell lines

Cell line source(s)	HEK293T (CRL-11268) and HeLa (CCL-2) were obtained from the American Type Culture Collection (ATCC).
Authentication	The cell lines were frequently checked by their morphological features and the cell lines are not been authenticated by the short tandem repeat (STR) profiling.
Mycoplasma contamination	All cell lines were tested to be mycoplasma-negative by the standard PCR method
Commonly misidentified lines (See ICLAC register)	No commonly misidentified cell lines are used in this study.

Animals and other organisms

Policy information about studies involving animals; ARRIVE guidelines recommended for reporting animal research

Laboratory animals	Postnatal 56- to 70-day-old (P56-70) wild-type C57BL/6N mice (male and female, random choice; purchased from Vital River Laboratories, China.) were used to prepare the acute brain slices and two-photon in vivo imaging. All mice were either family-housed or pair-housed in a temperature-controlled room (21.5 degree centigrade) with a 12-h/12-h light/dark cycle, with humidity controlled as 55%.
Wild animals	No wild animals are used in this study.

Field-collected samples	The study did not involve samples collected from the field.
Ethics oversight	All protocols were approved by the Institutional Animal Care and Use Committee of Peking University accredited by AAALAC International.

Note that full information on the approval of the study protocol must also be provided in the manuscript.

Flow Cytometry

Plots

Confirm that:

- [ ] The axis labels state the marker and fluorochrome used (e.g. CD4-FITC).
- [ ] The axis scales are clearly visible. Include numbers along axes only for bottom left plot of group (a 'group' is an analysis of identical markers).
- [ ] All plots are contour plots with outliers or pseudocolor plots.
- [ ] A numerical value for number of cells or percentage (with statistics) is provided.

Methodology

Sample preparation	For E. coli, an overnight E. coli BW25113 culture harboring both the hxlAB-gfp reporter plasmid and the pBAD-HxlR plasmid was inoculated (1:100) in LB medium and grown to an OD600nm of 0.6. L-Arabinose with 4 mM (~0.06% w/v) in final concentration was added to induce expression of HxlR protein. After 1 h induction, bacterial cells were next treated with or without 600 µM FA for 40 min before being analyzed by flow cytometry.<br><br>For cultured mammalian cells, HEK293T cells transfected with FAsor variants in a 24-well culture plate (Corning) were changed to fresh DMEM with different reagents. After incubation the cells were trypsinized and resuspended into 0.5 mL PBS for analysis by flow cytometry.
Instrument	BD LSRFortessa
Software	Collected by BD FACSDiva, and analyzed by FCS Express
Cell population abundance	Flow cytometry was used for quantification purposes only and no cell sorting was performed
Gating strategy	For all experiments FSC-A/ SSC-A gates of the starting cell population were used to discriminate between viable cells and cell debris.

- [x] Tick this box to confirm that a figure exemplifying the gating strategy is provided in the Supplementary Information.
